# Ammonia for post-healing of formamidinium-based Perovskite films

**DOI:** 10.1038/s41467-022-32047-z

**Published:** 2022-07-29

**Authors:** Zhipeng Li, Xiao Wang, Zaiwei Wang, Zhipeng Shao, Lianzheng Hao, Yi Rao, Chen Chen, Dachang Liu, Qiangqiang Zhao, Xiuhong Sun, Caiyun Gao, Bingqian Zhang, Xianzhao Wang, Li Wang, Guanglei Cui, Shuping Pang

**Affiliations:** 1 Qingdao Institute of Bioenergy and Bioprocess Technology, Chinese Academy of Sciences, Qingdao, 266101 P. R. China; 2 Center of Materials Science and Optoelectronics Engineering, University of Chinese Academy of Sciences, Beijing, 100049 P. R. China; 3 College of Materials Science and Engineering, Qingdao University of Science and Technology, Qingdao, 266042 P. R. China; 4 School of Future Technology, University of Chinese Academy of Sciences, Beijing, 100049 P. R. China

**Keywords:** Solar cells, Solar cells, Coordination chemistry

## Abstract

Solvents employed for perovskite film fabrication not only play important roles in dissolving the precursors but also participate in crystallization process. High boiling point aprotic solvents with O-donor ligands have been extensively studied, but the formation of a highly uniform halide perovskite film still requires the participation of additives or an additional step to accelerate the nucleation rate. The volatile aliphatic methylamine with both coordinating ligands and hydrogen protons as solvent or post-healing gas facilitates the process of methylamine-based perovskite films with high crystallinity, few defects, and easy large-scale fabrication as well. However, the attempt in formamidinium-containing perovskites is challenged heretofore. Here, we reveal that the degradation of formamidinium-containing perovskites in aliphatic amines environment results from the transimination reaction of formamidinium cation and aliphatic amines along with the formation of ammonia. Based on this mechanism, ammonia is selected as a post-healing gas for a highly uniform, compact formamidinium-based perovskite films. In particular, low temperature is proved to be crucial to enable formamidinium-based perovskite materials to absorb enough ammonia molecules and form a liquid intermediate state which is the key to eliminating voids in raw films. As a result, the champion perovskite solar cell based on ammonia post-healing achieves a power conversion efficiency of 23.21% with excellent reproducibility. Especially the module power conversion efficiency with 14 cm^2^ active area is over 20%. This ammonia post-healing treatment potentially makes it easier to upscale fabrication of highly efficient formamidinium-based devices.

## Abstract (web summary)

Solvents used for perovskite film fabrication not only dissolve the precursors but also play a role in the crystallization process. Here, authors study the role of transamination reactions in the underlying degradation mechanism of formamidinium-containing perovskites in aliphatic amines environment.

## Introduction

Halide perovskite materials with the general formula ABX_3_, where A refers to a monovalent cation such as methylammonium (CH_3_NH_3_^+^, MA^+^), formamidinium (HC(NH_2_)_2_^+^, FA^+^), Cs^+^, B represents a divalent cation such as Pb^2+^, Sn^2+^, Ge^2+^, and X represents a halide ion such as I^-^, Br^-^, Cl^-^, have emerged as a class of promising light-harvesting materials in photovoltaics since 2009^1^. The highest certified power conversion efficiency (PCE) of perovskite solar cells (PSCs) has so far been up to 25.7% ^2^, which is comparable to that of widely commercialized Si-based solar cells. The crystal structure of halide perovskites can be regarded as a [MX_6_]^4−^ octahedron in a three-dimensional (3D) space with a common apex angle connecting. The A site ions fill up the gap of the octahedral and the structure is stabilized by Van der Waals forces^3^. These special structural characteristics provide a variety of solution processing methods to prepare perovskite films^4–6^.

The high boiling point aprotic *N,N*-dimethylformamide (DMF), *γ*-butyrolactone (GBL), dimethyl sulfoxide (DMSO) and *N,N*-dimethylacetamide (DMA) are the common solvents employed to dissolve the perovskite precursors with the formation of solvated iodoplumbate complexes because of the strong coordination between O-donor ligands (OLs) and Pb(II)^4,7–9^. The Lewis basicity of solvents is thought to correlate with the “coordinating ability” with lead halide salts, which can be predicted by their Gutmann’s Donor Number (D_N_) with a trend of DMSO > DMA > DMF > GBL^10,11^. High D_N_ solvents with strong coordination with the Pb(II) center also result in the formation of intermediate phase OL-PbI_2_-RAI (RA refers to MA or FA, etc.) prior to perovskite phase during the film fabrication process. The anisotropic growth nature of the intermediate phase leads to rough films with one-dimensional (1D) fiber-like structures and a large proportion of void area^12^. In this regard, stronger coordinating additives (such as thiourea^13^ and pyridine^14^) and/or the fast nucleation strategies (such as anti-solvent extraction, gas-quenching, and vacuum-assisted drying) have been introduced to regulate the growth process of the intermediate phase to achieve highly uniform perovskite films with good crystallinity^7,15,16^.

Among them, methylamine (MA^0^) featured with the presence of hydrogen bonding and low boiling point has become an impressive coordination agent for the MAPbI_3_ material. In this case, the formed intermediate phase is a metastable (PbI_2_-MAI)·xMA^0^ complex, instead of simple coordinate bond-dominated OL-PbI_2_-MAI adducts. Easy formation of highly uniform (PbI_2_-MAI)·xMA^0^ films and following conversion to highly uniform perovskite films are closely related to the self-leveling behavior of (PbI_2_-MAI)·xMA^0^ liquid intermediate phase and the ultrafast evaporation of MA^0^ gas. MA^0^ employed as a post-healing gas to eliminate the voids in the MAPbI_3_ perovskite film is firstly reported by Zhou et al. in 2015^17^, and the adoption of MA^0^ as a volatile solvent system has become a commercially viable technology for MAPbI_3_ devices with excellent device reproducibility^18–23^. In comparison with MAPbI_3_, α-FAPbI_3_ has higher theoretical efficiency and thermal stability. The undesired phase transition to non-perovskite phase δ-FAPbI_3_ has been well dissolved by alloying a small amount of Cs^+^, MA^+^ to tailor the tolerance factor^24–29^. We have attempted to introduce MA^0^ gas-related methods for the fabrication of FA-containing perovskite layers, unfortunately, resulting in degradation of the 3D perovskite phase^18^. The underlying reason for the irreversible transformation of FAPbI_3_ with MA^0^ treatment is still not fully clear, let alone make efforts to solve this problem.

Here, we have systematically studied the underlying chemical reactions between aliphatic amines/formamidine (FA^0^) gases and FAI salt, and elucidated the addition-elimination reaction between amine compound and the imine band of FA^0^ with the formation of ammonia (NH_3_), also named transimination reaction. Based on this mechanism, NH_3_ is selected as a post-healing gas to avoid the degradation of the FA-based perovskite phase during the post treatment. It is demonstrated that decreasing processing temperature is crucial for FA-based perovskite layer to enhance the absorption of NH_3_ molecules, leading to the formation of a desired liquid intermediate state. Self-leveling behavior of the liquid intermediate state can quickly heal the voids in the rough perovskite film and finally form a highly uniform and compact film with the evaporation of NH_3_. At last, the PCE of FA-based PSC based on this NH_3_ post-healing strategy is more than 23% with a certified PCE of 22.22%. Especially, the PSC module achieves a PCE of 20.61% which is comparable with the highest reports in PSC modules. These results demonstrate the large advantage of the NH_3_ gas post-healing technology in upscaling fabrication of highly efficient PSCs.

## Results

### The degradation evidence of formamidinium-containing perovskites in aliphatic amines environment

Nuclear magnetic resonance (NMR) spectroscopy is a particularly practical tool for quantifying the relative amounts of the organic cations and related chemical reactions in the perovskite precursor^30–33^. We started with the study of the underlying chemical reactions between FA^0^/R-NH_2_(RA^0^) gases and FAI salt (Table 1). The gases involved include FA^0^, MA^0^, ethylamine (EA^0^), *n*-propylamine (PA^0^), *n*-butylamine (BA^0^), and NH_3_. The detailed preparation procedures of the relevant gases are provided in the methods section.

**Table 1:** The synthesis of formamidine (FA^0^) and chemical reactions between FA^0^/amines/NH_3_ gases and FAI salt

No.	Gas generation	Gas collection	Product^a^
1	FACl, NaOH, RT	DMSO-d_6_	NH_3_
2	FACl, NaOH, 150 °C	DMSO-d_6_	*s*-triazine, formamide, FA, NH_3_
3	FACl, NaOH, 150 °C	HOAc	FAAc^b^
4	FACl, NaOH, 150 °C	FAI powder	FAI, formamide^b^
5	MA in EtOH, 60 °C	FAI powder	DMFAI^b^
6	EACl, NaOH, 60 °C	FAI powder	DEFAI^b^
7	PA, 60 °C	FAI powder	DPFAI^b^
8	BA, 60 C	FAI powder	DBFAI^b^
9	NH_3_·H_2_O, 60 °C	FAI powder	NH_4_I^b^
10	MA in H_2_O, 60 °C	FAI powder	MAI^b^
11	EA in H_2_O, 60 °C	FAI powder	EAI^b^
12	H_2_O, 60 °C	FAI powder	FAI^b^
13	NH_3_, RT	FAI powder	FAI^b^

^a^ The main components are listed.

^b^ The samples are treated with vacuum to remove low boiling point components before ^1^H NMR measurement. FACl is formamidine hydrochloride, FA is formamidine, HOAc is acetic acid, FAAc is formamidinium acetic, FAI is formamidinium iodide, MA is methylamine, EtOH is ethyl alcohol, DMFAI is *N,N’*-dimethyl formamidinium iodide, EACl is ethylamine hydrochloride, DEFAI is *N,N’*- diethyl formamidinium iodide, PA is *n*-Propylamine, DPFAI is *N,N’*- dipropyl formamidinium iodide, BA is *n*-Butylamine, DBFAI is *N,N’*- dibutyl formamidinium iodide. MAI is methylaminium iodide, and EAI is ethylamine hydroiodide.

The synthesis of FA^0^ is tricky because of its instability. Mixed FACl and NaOH powders are expected to form FA^0^ by the neutralization as shown in Supplementary Scheme 1a. To study their product, deuterated DMSO (DMSO-d_6_) solvent is used to collect released gas from the mixed powders at room temperature (RT) by a simple equipment (Supplementary Fig. 1a) for ^1^H nuclear magnetic resonance (^1^H NMR) measurement. Only NH_3_ signal is detected in the ^1^H NMR (Table 1 (No. 1) and Supplementary Fig. 4). We think that the high boiling point of FA^0^ likely makes FA^0^ molecules difficult to diffuse into DMSO-d_6_ solvent. The formation of NH_3_ is indicative of the existence of some chemical reactions in the process of gas preparation (Supplementary Fig. 2). When the temperature of the reactor and the gas pipeline is increased to 150 °C and DMSO-d_6_ solvent is still kept at RT, four main compounds are detected: *s*-triazine, formamide, FA^0^, and NH_3_ (Table 1 (No. 2) and Supplementary Fig. 5). The formation of *s*-triazine is because of the transimination reaction of three FA^0^ molecules along with the formation of NH_3_ (Supplementary Fig. 2b)^30,31^. The *s*-triazine has also been detected when the FAI powder is heated at 150 °C^32^. Formamide is attributed to the hydrolyzation product of FA^0^ (Supplementary Fig. 2c) in the presence of H_2_O which is generated from the neutralization between FACl and NaOH. Considering the long-time interval from gas collection to ^1^H NMR measurement, we then employ acetic acid (HOAc) to capture FA^0^ so as to limit these chemical reactions in DMSO-d_6_ solvent by the quick formation of FAAc. As expected, ^1^H NMR spectrum verifies that the reaction product at 150 °C only exists FAAc rather than *s*-triazine, formamide, or NH_4_Ac (Table 1 (No. 3) and Supplementary Fig. 6). The above results strongly indicate the formation and instability of FA^0^. When FAI powder is exposed to FA^0^, there is hardly any change in chemical compositions except the formation of a tiny amount of formamide as the presence of H_2_O (Table 1 (No. 4), Supplementary Fig. 7). These spontaneous reactions and instability of FA^0^ seriously limit its practical application in post-healing FA-based perovskite films.

In comparison with FA^0^, amines are more stable. When FAI powder is treated with MA^0^, EA^0^, PA^0^, or BA^0^, the powder quickly transforms into a liquid state. After the liquid is held at 60 °C for 6 h and then vacuumed (Supplementary Fig. 1b), it is unexpected that there are no FAI signals in ^1^H NMR spectrum of the final powder samples (Table 1 (No. 5–8), Supplementary Figs. 8–11).

**Fig. 1:**
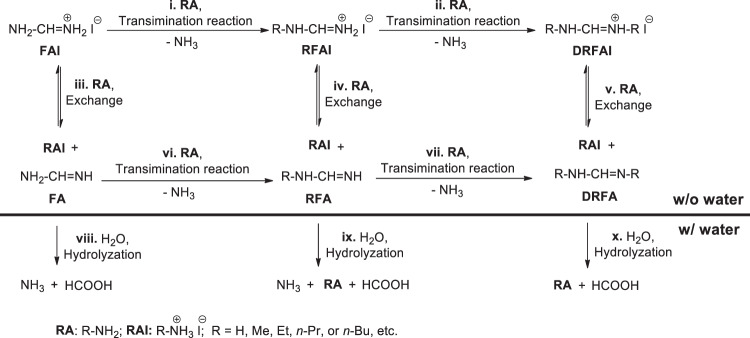
Chemical reactions related to FAI. The transimination reactions and the ion exchange reactions between FAI and RA^0^ molecules (R refers to H, Me, Et, *n*-Pr, or *n*-Bu etc.), and the hydrolysis reactions of FA^0^, RFA^0^, and DRFA^0^.

Taking MA^0^ as an example (Table 1 (No. 5)), we find that the main signals in ^1^H NMR spectrum belong to *N,N*’-dimethyl formamidinium iodide (DMFAI) (Supplementary Figs. 3 and 8)^33^. The related chemical reactions are illustrated in Fig. 1 (i, ii). The lone-pair electrons of N atom in nucleophilic MA^0^ molecule can attack electrophilic imine bond in FAI, which leads to the formation of *N*-methyl formamidinium iodide (MFAI) by a transimination process (Fig. 1(i), Supplementary Fig. 3)^34^. The imine bond in the formed MFAI can carry out the second transimination reaction with MA^0^ to form DMFAI (Fig. 1(ii), Supplementary Fig. 3). With excessive MA^0^ and holding for enough time, FAI can fully transform into DMFAI. Similarly, when FAI powders are treated with EA^0^, PA^0^ and BA^0^ gases, the transimination reaction also occur with the formation of DEFAI, DPFAI and DBFAI, respectively (Table 1 (No. 6–8), Supplementary Figs. 9–11, Fig. 1(i, ii)).

When FAI powder is treated by RA^0^ (R is referred to H, Me, Et etc.) gas with some H_2_O vapor evaporated from their aqueous solution (Supplementary Fig. 1c), the final product is RAI rather than FAI, RFAI or DRFAI (Table 1 (No. 9–11), Supplementary Figs. 12–14). Considering there is no reaction between FAI salt and H_2_O (Table 1 (No. 12), Supplementary Fig. 15), the formation of RAI mentioned above is due to the reversible ion exchange reactions between FAI, RFAI or DRFAI and RA^0^ with the formation of RAI and FA^0^, RFA^0^, or DRFA^0^. The hydrolysis reactions of FA^0^, RFA^0^, and DRFA^0^ lead to the formation of volatile HCOOH and RA^0^, which in turn promotes the ion exchange reactions towards the formation of RAI (Fig. 1 (iii–x))^35^.

Supplementary Fig. 16 shows the optical photos and XRD patterns of α-FAPbI_3_ films before and after MA^0^ post-healing treatment. δ-FAPbI_3_, MFAPbI_3_, and DMFAPbI_3_ films are also measured for comparison. The formation of the non-perovskite phase after MA^0^ post-healing the α-FAPbI_3_ film is because of the conversion from FA^+^ to MFA^+^ and DMFA^+^. This is why aliphatic amines cannot be employed as solvents or post-healing gases for the fabrication of FA-containing perovskite materials. Inspired by the above transimination reaction, NH_3_ is selected to avoid the change of composition after the gas post-healing treatment. The ^1^H NMR spectrum well verifies no composition change of FAI powder after being treated with NH_3_ gas (Table 1 (No. 13), Supplementary Fig. 17).

### Feasibility analysis of ammonia as the healing gas

**Fig. 2:**
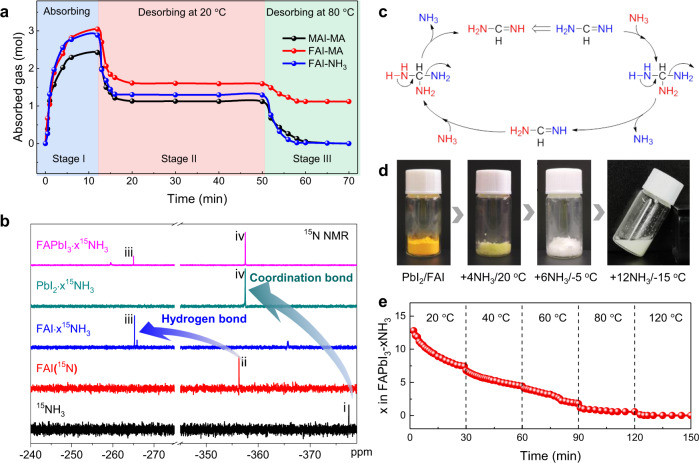
The interaction of NH_3_ with FA-based perovskite or precursors. **a** Absorption and desorption behavior of MAI-MA^0^, FAI-MA^0^, and FAI-NH_3_. **b** The solution ^15^N NMR spectra of ^15^NH_3_, FAI(^15^N), FAI·x^15^NH_3_, PbI_2_·x^15^NH_3_, and FAPbI_3_·x^15^NH_3_. Signals: i, ^15^N signal in ^15^NH_3_; ii, ^15^N signal in FAI (^15^N); iii, ^15^N signal in FAI hydrogen bonded with ^15^NH_3_; iv, ^15^N signal of ^15^NH_3_ coordinated with Pb(II). **c** Reaction mechanism of transimination reactions between NH_3_ and FAI. **d** The photographs of PbI_2_/FAI powder in NH_3_ atmosphere under different temperatures. **e** The calculated *x* value of FAPbI_3_·xNH_3_ complex in an open condition under different temperatures.

The reversible absorption-desorption process of gas is crucial for the post-healing strategy. MAI and FAI samples are weighted during MA^0^/NH_3_ absorption and desorption as shown in Fig. 2a. The whole process can be divided into gas absorption at RT (Stage I), desorption at RT (Stage II), and desorption at 80 °C (Stage III). When exposed to MA^0^, MAI quickly absorbs MA^0^ and reaches a saturated state with the *x* value of about 2.5 in MAI·xMA^0^ (the *x* value is very sensitive to temperature and pressure). The liquid MAI·xMA^0^ complex removed from MA^0^ atmosphere desorbs MA^0^ gas spontaneously until it transforms into a relative stable semi-solid-state with the *x* value of about 1.1, which is denoted as stage II. After further heating at 80 °C for 10 min (Stage III), the sample returns to a white powder with the same weight as the initial MAI, which means the complete reversibility of absorption-desorption process in MAI-MA^0^ pair. In FAI-MA^0^ pair, the Stage I and Stage II are similar to these of MAI-MA^0^ pair, but the weight of the final powder in the Stage III cannot return back to the initial value of FAI because of the formation of MFAI and DMFAI as presented in Fig. 2a.

FAI powder exposed to NH_3_ gas atmosphere rapidly achieves a liquid phase FAI·xNH_3_ (Supplementary Fig. 18) with the x value of ∼3.0 (Stage I). The liquid phase transforms into an ice-like transparent solid-state FAI·xNH_3_ with *x* ∼ 1.3 as NH_3_ desorption in Stage II and the XRD pattern of the ice-like complex is shown in Supplementary Fig. 17. The following thermal annealing at 80 °C fully removes the absorbed NH_3_ molecules and leads to the formation of a white powder with the same weight as the initial FAI (Stage III). This phenomenon, similar to that of MAI-MA^0^ pair, preliminarily indicates the feasibility of the NH_3_ as a post-healing gas for FA-based perovskite films.

To make a profound study, the chemical reaction and intermolecular interactions between FAPbI_3_ precursors and NH_3_ molecules, the solution ^15^N NMR spectra are measured as shown in Fig. 2b and Supplementary Figs. 19–24. The measured isotopically labeled ^15^N samples are ^15^NH_3_, FAI treated with ^15^NH_3_, and vacuumed (FAI(^15^N)), FAI absorbing ^15^NH_3_ molecules (FAI·x^15^NH_3_), PbI_2_ absorbing ^15^NH_3_ molecules (PbI_2_·x^15^NH_3_), and FAPbI_3_ absorbing ^15^NH_3_ molecules (FAPbI_3_·x^15^NH_3_). The ^15^N chemical shift of ^15^NH_3_ in DMSO-d_6_ is −377.46 ppm. The ^15^N NMR spectrum of FAI after treatment with ^15^NH_3_ (FAI (^15^N)) shows the ^15^N signal at −356.33 ppm, which indicates the existence of transimination reaction between NH_3_ and FAI (Supplementary Fig. 3). The reaction process between the NH_3_ and FAI is similar to the transimination reaction involving the aliphatic amine. The lone-pair electrons of N atom in NH_3_ can attack electrophilic imine bond in FAI, leading to the exchange of ^15^N in NH_3_ and ^14^N in FAI and formation of FAI(^15^N), as shown in Fig. 2c. Besides, the ^15^N chemical shift of FAI in FAI ·x^15^NH_3_ is at −265.22 ppm, which moves towards the low field about 91 ppm in comparison with FAI(^15^N). It suggests the presence of hydrogen bond interaction between NH_3_ and FAI. Furthermore, the ^15^N chemical shift of PbI_2_·x^15^NH_3_ at −357.51 ppm moves towards the low field about 20 ppm compared to that of ^15^NH_3_, which is due to the coordination of ^15^NH_3_ to Pb(II). The ^15^N chemical shifts of FAPbI_3_·x^15^NH_3_ at −265.02 ppm and −357.53 ppm correspond to that of FA^+^(^15^N) with hydrogen bond interaction with NH_3_ and that of ^15^NH_3_ with coordination bond interaction with Pb(II), respectively. The coexistence of hydrogen bond and coordination bond in the intermediate phase FAPbI_3_·x^15^NH_3_ is also supported by the solid-state ^15^N NMR result in Supplementary Fig. 25.

NH_3_, employed as a post-healing gas, should also have good solubility for the FA-based perovskite precursors, including PbI_2_ and FAI powders. The color of mixed PbI_2_/FAI powder exposed in NH_3_ gas atmosphere turns into light yellow from the yellow of PbI_2_ at 20 °C^36^. The mass difference before and after absorption shows that the absorbed NH_3_ molecule number per FAPbI_3_ is ∼4. While FAPbI_3_·4NH_3_ is still a solid-state rather than an expected liquid state, which is very different from liquid MAPbI_3_·xMA with x of only 3^17^. One possible reason is the much smaller size of NH_3_ than MA^0^. We have found that the absorbed amount of gas is highly dependent on processing temperature^23^. Lowering the processing temperature to −5 °C leads to the formation of a white powder with *x* value of ∼6. At −15 °C, a flowable slurry with the *x* value of ∼12 is formed, as shown in Fig. 2d.

The kinetic process of NH_3_ desorption from the flowable FAPbI_3_·xNH_3_ complex is measured by monitoring its weight change with temperature increased step-by-step from 20 °C to 120 °C under an open condition. The absorbing NH_3_ number per FAPbI_3_ as the function of time illustrated in Fig. 2e shows that the weight of FAPbI_3_·xNH_3_ slurry drops rapidly at the initial stage and then decreases slowly in 30 min at 20 °C. Then the sample was annealed at 40 °C for 30 min and 60 °C for 30 min showing continuously slow weight loss by weighing at regular intervals. At 80 °C, the sample quickly reaches a relatively stable state then almost no weight loss in the maintained 30 min and the NH_3_ molecular number per FAPbI_3_ is about 1.0. Finally, the *x* value quickly decreases to 0 when the sample is annealed at 120 C.

### Preparation and characterization of thin films

**Fig. 3:**
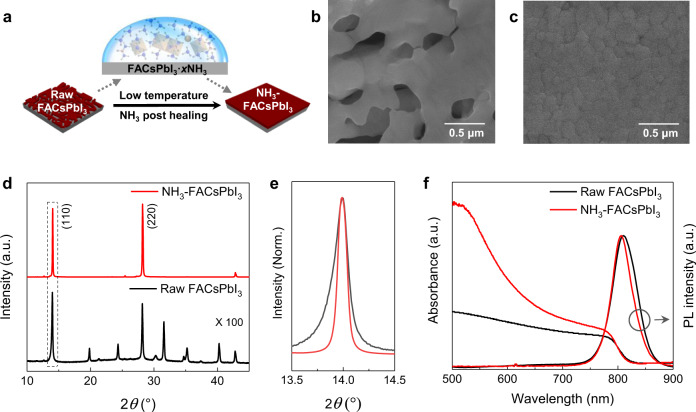
Properties of perovskite Films. **a** Schematic illustration of NH_3_ post-healing FACsPbI_3_ perovskite thin film. **b**, **c** Scanning electron microscope (SEM) images of (b) raw FACsPbI_3_ and (c)NH_3_-FACsPbI_3_ perovskite films. **d**, **e** X-ray diffraction (XRD) patterns, **f** Ultraviolet-visible (UV-Vis) spectra and steady photoluminescence (PL) spectra of raw FACsPbI_3_ and NH_3_-FACsPbI_3_ perovskite films, respectively.

Then the NH_3_ post-healing strategy is employed to prepare FA-based perovskite films based on a homemade chamber with a semiconductor chilling plate controlling chamber temperature (Supplementary Fig. 26). As shown in Supplementary Fig. 27a, b, the raw FAPbI_3_ film with voids by the traditional one-step spin-coating method can transform into a uniform film after NH_3_ post-healing at −15  °C. The NH_3_ post-healing FAPbI_3_ is denoted as NH_3_-FAPbI_3_. The XRD spectra show that the NH_3_-FAPbI_3_ film has better crystallinity and less undesired δ phase than the raw film (Supplementary Fig. 27c). This leads to the increasing PCE of the NH_3_-FAPbI_3_ device compared to the raw-FAPbI_3_ device (Supplementary Fig. 27d). While the PCE of NH_3_-FAPbI_3_ device is still limited due to the existence of undesired δ-phase in the perovskite film. To prepare highly efficient PSCs, cesium (Cs) doped FAPbI_3_ material system FA_0.9_Cs_0.1_PbI_3_ (FACsPbI_3_) is selected. The NH_3_ post-healing process of FACsPbI_3_ film is schematically illustrated in Fig. 3a. Due to the thin thickness of perovskite films, the NH_3_ gas absorption and desorption are much easier and faster than those of powder samples. The SEM images show that the raw FACsPbI_3_ film could be well healed by the NH_3_ post-healing method in the temperature range from −15 °C to 0 °C, and −15  °C was used for NH_3_ gas post-healing in the following study (Supplementary Fig. 28).

The scanning electron microscope (SEM) image (Fig. 3b) shows that there are some micrometer-scale voids in the raw FACsPbI_3_ film. While the NH_3_-FACsPbI_3_ film is dense and smooth (Fig. 3c), resulting from the self-leveling of the liquid intermediate state and the quick desorbing process of the NH_3_. This gas post-healing treatment with the self-leveling behavior is essentially different from the traditional post-treatment using DMF^37^, MASCN vapor^25^, etc. The corresponding atomic force microscope (AFM) images (Supplementary Fig. 29) show that the root mean square (RMS) roughness of NH_3_-FACsPbI_3_ film is only 9 nm over a 20 × 20 µm^2^ area, which is even lower than that of the film prepared by the traditional antisolvent method (24 nm) (denoted as anti-FACsPbI_3_).

X-ray diffraction (XRD) patterns in Fig. 3d and e show that the NH_3_ post-healing strategy can improve the orientation of the perovskite film, and the diffraction intensity of (110) peak of the NH_3_-FACsPbI_3_ perovskite film is about 100 times stronger than that of the raw FACsPbI_3_ perovskite film. Besides, NH_3_ post-healing decreases the full-width half-maximum (FWHM) of the (110) peak from 0.203^o^ to 0.114^o^ (Fig. 3e). As shown in ultraviolet-visible (UV-Vis) optical absorption spectra (Fig. 3f) and their Tauc plot curves (Supplementary Fig. 30), the raw and NH_3_-FACsPbI_3_ perovskite films present almost the same absorption edge and band gap of 1.54 eV. While the absorbance of NH_3_-FACsPbI_3_ perovskite film exhibits an obvious increase compared to that of raw perovskite film, resulting from the removal of voids. Photoluminescence (PL) peak of the NH_3_-FACsPbI_3_ film is similar to that of the anti-FACsPbI_3_ film (Supplementary Fig. 31), but obviously blue shift and narrow in comparison with the raw FACsPbI_3_ film, which indicates a low trap density of NH_3_-FACsPbI_3_ and anti-FACsPbI_3_ films in comparison with the raw film^38^. The reduced defect density is also evidenced by the charge-limited current (SCLC) and time-resolved photoluminescence (TRPL) results in Supplementary Fig. 32. The calculated defect density from the SCLC curves decreases from 3.97 × 10^16^ to 9.62 × 10^15^ cm^−3^. And the TRPL results show that the NH_3_-FACsPbI_3_ film has a much longer trap-assisted nonradiative lifetime (τ = 5.6 μs) than the Raw FACsPbI_3_ film (τ = 3.2 μs).

### Device performance evaluation

**Fig. 4:**
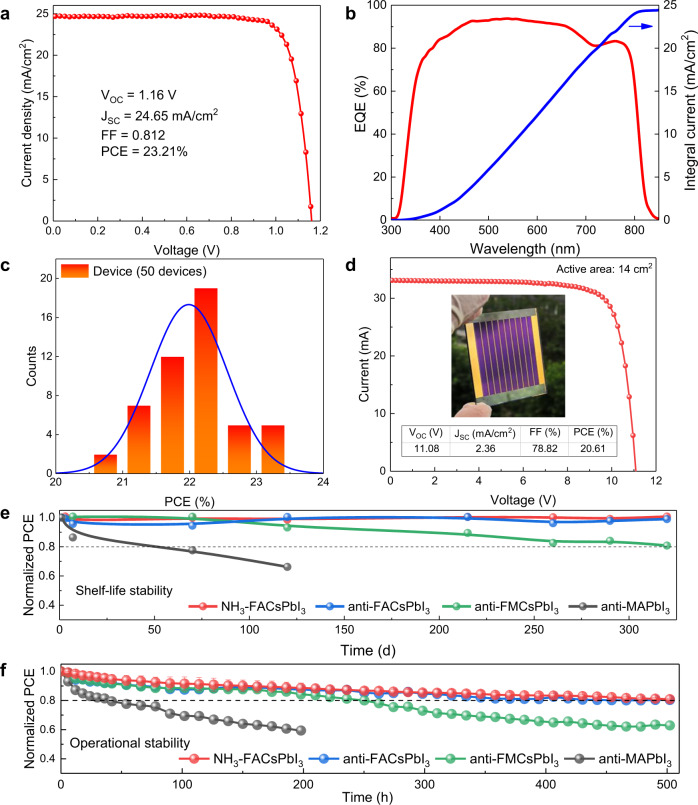
Device performance and stability. **a** Current density–voltage (*J-V*) curve of the champion PSCs based on NH_3_-FACsPbI_3_. **b** External quantum efficiency (EQE, red) and the integrated short-circuit current density (blue) of the champion NH_3_-FACsPbI_3_ device. **c** Histogram of the power conversion efficiency for 50 NH_3_-FACsPbI_3_ PSCs. **d** *J-V* curve of NH_3_-FACsPbI_3_ PSC module. **e** Normalized power conversion efficiency (PCE) of unencapsulated NH_3_-FACsPbI_3_, anti-FACsPbI_3_, anti-FMCsPbI_3,_ and anti-MAPbI_3_ devices with the storage time of 320 days under 10–30% relative humidity at room temperature. **f** Evolution of the normalized PCE over time measured by maximum power point tracking of unencapsulated NH_3_-FACsPbI_3_, anti-FACsPbI_3_, anti-FMCsPbI_3,_ and anti-MAPbI_3_ devices under light soaking with full solar intensity. Standard deviation (error bar) was calculated from three individual devices in the same batch.

The planar devices with a configuration of FTO/TiO_2_-SnO_2_/perovskite/2,2′,7,7′-tetrakis(*N,N*-di-p-methoxyphenyl-amine)−9,9′-spirobifluorene (Spiro-OMeTAD)/Au are fabricated. The champion solar cell (Fig. 4a) based on NH_3_-FACsPbI_3_ film displays a PCE of 23.21%, with open-circuit voltage (*V*_OC_) of 1.16 V, short-circuit current density (*J*_SC_) of 24.65 mA/cm^2^, fill factor (FF) of 81.20%, demonstrating obvious improvement in comparison with the PCE of 10.92% of the control device based on raw FACsPbI_3_ film and comparable performance compared to the devices (PCE = 22.99%) based on the anti-FACsPbI_3_ film in our lab (Supplementary Fig. 33). The certified PCE of NH_3_-FACsPbI_3_ PSC is 22.22% (Supplementary Fig. 34). Meanwhile, there is less hysteresis for the NH_3_-FACsPbI_3_ device (Supplementary Fig. 35). The stabilized power output (SPO) of NH_3_-FACsPbI_3_ champion cells is 22.93%, shown in Supplementary Fig. 36. The *J*_SC_ of NH_3_-FACsPbI_3_ PSC is comparable with the integrated *J*_SC_ from EQE results (24.41 mA/cm^2^) in Fig. 4b. The distribution histogram of PCE based on 50 NH_3_-FACsPbI_3_ devices at reverse scan direction (Fig. 4c, Supplementary Table 1) shows excellent reproducibility of high-performance NH_3_ post-healing devices. The cross-sectional SEM image of NH_3_-FACsPbI_3_ PSC shows the uniform thickness and good interfacial contact of perovskite film with transport layers (Supplementary Fig. 37). Combined NH_3_ gas healing and the doctor-blading technology (Suzhou GCL Nano Co. Ltd.), a prototype PSC module consisting 10 cells in series connection achieves a PCE of 20.61% (Fig. 4d) and the certified PCE of the module is 19.38% (Supplementary Fig. 38) with an estimated active area of 14.00 cm^2^, which is comparable with the reported highest efficiencies in perovskite modules^39–41^.

To further evaluate the device stability based on NH_3_ post-healing treatment, the high-efficiency NH_3_-FACsPbI_3_, anti-FACsPbI_3_, anti-FA_0.90_Cs_0.05_MA_0.05_PbI_3_ (anti-FMCsPbI_3_), and anti-MAPbI_3_ PSCs are employed. As the low PCE, raw FACsPbI_3_ device has not been chosen to study its stability. The shelf stability of unencapsulated devices in Fig. 4e shows that NH_3_-FACsPbI_3_ device has negligible performance loss after 320 days of storage, which is similar to the anti-FACsPbI_3_ devices and much better than anti-FMCsPbI_3_ and anti-MAPbI_3_. The XRD and UV-Vis spectra of these aged perovskite films in 320-day-aged PSCs (Supplementary Figs. 39–41) show that NH_3_-FACsPbI_3_ film keeps its strong light absorption, and has few PbI_2_. Meanwhile, NH_3_-FACsPbI_3_ device still maintains decent cross-sectional morphology with clear grain boundaries (Supplementary Fig. 42). What’s more, the maximum power point (MPP) of the unencapsulated NH_3_-FACsPbI_3_, anti-FACsPbI_3_, anti-FMCsPbI_3,_ and anti-MAPbI_3_ PSCs is tracked under white light emitting diode (LED) irradiation (Fig. 4f, Supplementary Fig. 43) with an intensity equivalent to 1 sun in N_2_ atmosphere. After 500 h of continuous light soaking, the NH_3_-FACsPbI_3_ device maintains 80% of its initial efficiency, and the stability trend of MPP tracking is consistent with that of shelf stability in these devices.

## Discussion

We have systematically studied the transimination reactions between FA/RA gases and FAI salt, and further developed an NH_3_ gas post-healing strategy for upscale fabrication of high-quality FA-based perovskite films. The low operating temperature during NH_3_ post-healing process is vital to enable FA-based perovskite materials to absorb enough NH_3_ molecules and transform into a flowable intermediate state. Based on this strategy, the champion device achieves a PCE of 23.21% (Certified PCE of 22.22%), and the PCE of the perovskite module is up to 20.61%. Meanwhile, the device stability based on this strategy is also comparable with that of the state-of-the-art anti-solvent method. This NH_3_ gas post-healing technology is compatible with established commercial technologies to efficiently remove voids in the raw FA-based films and therefore opens a direction for fabrication of large-scale high-efficient FA-based PSCs.

## Methods

### Materials

*N,N*-dimethylformamide (DMF, anhydrous 99.8%), dimethyl sulfoxide (DMSO, anhydrous 99.9%) were procured from Sigma-Aldrich. Formamidine hydrochloride (FACl, 96%), Ethylamine Hydrochloride (EACl, 98%), *n*-Propylamine, (PA, 99%), *n*-Butylamine (BA, 98%), and Lead iodide (PbI_2_, >98.0%) was purchased from TCI. Formamidinium iodide (FAI, 99.5%), Methanaminium iodine (MAI, 99.5%), Cesium iodide (CsI, 99.99%), and Spiro-OMeTAD (99.8%) were purchased from Xi’an Polymer Light Technology Corp. (PLT). Ammonium chloride (^15^NH_4_Cl, 10 atom% ^15^N labelled, ≥98.5%) was purchased from Macklin. Ammonium chloride (^15^NH_4_Cl, 99 atom% ^15^N labelled, >98%) and DMSO-d_6_ (99 atom% D labelled) were purchased from Cambridge Isotope Laboratories, Inc. (CIL). Sodium hydroxide (NaOH, 96%), Methylamine alcohol solution (30–33 wt. % in anhydrous ethanol), and Ammonium Hydroxide (NH_3_ ∙ H_2_O, 25∼28%) were purchased from Sinopharm Chemical Reagent Co., Ltd. The above chemicals were used as received without any purification.

### Material synthesis

For the synthesis of N-methyl formamidinium iodide (MFAI), Methylamine alcohol solution (30–33 wt. % in anhydrous ethanol, 425 μl, about 2.8 mmol) was added dropwise to a solution of formamidinium iodide (FAI, 516 mg, 3 mmol) in 30 ml anhydrous ethanol under nitrogen protection, and the mixture was stirred at 0 °C for 2 h. Subsequently, the solvent was evaporated under reduced pressure and the residue was recrystallized in the mix solvent of isopropanol and ethyl acetate in glove box to obtain the MFAI powder. The synthesis of N, N’-dimethyl formamidinium iodide (DMFAI) was similar to that of MFAI, but the amount of the methylamine alcohol solution was increased to 1 ml.

### NMR sample fabrication

For solution ^1^H NMR characterization of the sample in Table 1 (No.1), the mixture of 5.0 g FACl and 5.0 g NaOH was reacted at RT in a conical flask, and the generated gas was collected by 0.5 ml DMSO-d_6_. For samples in Table 1 (No. 2–4), the mixture of 5.0 g FACl and 5.0 g NaOH was reacted at 150 °C, and the generated gas was collected by 0.5 ml DMSO-d_6_, 1 ml HOAc or 0.2 g FAI powder at RT. Samples No. 3 and 4 were vacuumed before dissolving in DMSO-d_6_. For samples in Table 1 (No. 5–12), the gas sources of R-NH_2_ were heated at 60 °C, and the generated gases were collected by 0.2 g FAI powder. The gas sources of R-NH_2_ were methylamine alcohol solution, the mixture of EACl and NaOH, PA, BA, NH_3_·H_2_O, MA aqueous solution, EA aqueous solution, or H_2_O, respectively. The samples (No. 5–12) were kept at 60 °C for 6 h, and then vacuum treated to remove the low boiling point components. For the sample in Table 1 (No. 13), the NH_3_ gas was collected with 0.2 g FAI powder. The sample was kept at RT for 6 h and then vacuumed. The obtained solid powders in Table 1 (No. 3–13) were dissolved in DMSO-d_6_ for ^1^H NMR characterization.

For solution ^15^N NMR characterization, ^15^NH_3_ was produced from the reaction between 5.0 g ^15^NH_4_Cl (10% ^15^N labelled) and 5.0 g NaOH at 60 °C, and the generated gas was collected by 0.5 ml DMSO-d_6_. FAI (^15^N) was produced based on the reaction between 0.2 g FAI and excess ^15^NH_3_ at RT for 6 h, followed by vacuum treatment. FAI·x^15^NH_3_, PbI_2_·x^15^NH_3_, and FAPbI_3_·x^15^NH_3_ were generated between the reaction between 0.2 g FAI, 0.2 g PbI_2_, or 0.2 g FAPbI_3_ precursor powders and excess ^15^NH_3_ at RT for 6 h, but without vacuum treatment. The above samples were directly dissolved in DMSO-d_6_ for ^15^N NMR measurement.

The NMR sample fabrication of FAPbI_3_·x^15^NH_3_ for the solid-state NMR spectrum was similar to that of the solution ^15^N NMR spectrum, but ^15^N isotope source for preparing ^15^NH_3_ was used ^15^NH_4_Cl (99% ^15^N labelled) as the requirement of high ^15^N concentration for solid-state NMR.

### NMR measurements

The solution ^1^H NMR spectra were measured with a Bruker AVANCE III 600 instrument operating at 600.13 MHz at 298 K. Chemical shifts d (ppm) were referenced to the internal solvent signals.

The solution ^1^H-decoupled ^15^N NMR spectra (pules program zgig) were acquired using a Bruker Avance III 600 instrument operating at 600.13 MHz for ^1^H and 60.82 MHz for ^15^N. The ^15^N chemical shifts were determined from 1 M urea in DMSO (−303.20 ppm, 10% ^15^N labelled) as external standard reference 42. The relaxation delay was 10 s, the acquisition time was 1.3 s, and 128 scans were accumulated.

The solid-state NMR experiment was performed on a Bruker AVNCE 400 spectrometer operating at 399.95 MHz for ^1^H and 40.54 MHz for ^15^N. The obtained sample was packed into a 7 mm ZrO_2_ rotor. The ^15^N chemical shifts were determined from the ^15^NH_4_Cl (−341.17 ppm, 10% ^15^N labelled)^43^. The HPEDC pulse sequence was used. The acquisition time was 50 ms, the spin rate was 3 kHz, and the recycle delay was 5 s. The two-pulse phase-modulation (TPPM) decoupling was used during the acquisition.

### Perovskite film fabrication

Raw FACsPbI_3_ films: The perovskite precursor was prepared by mixing PbI_2_: FAI: CsI (1:0.9:0.1 molar ratio) in DMF solvent (1.40 M). Raw FAPbI_3_ films: The perovskite precursor was prepared by mixing PbI_2_: FAI (1:1 molar ratio) in DMF solvent (1.40 M). The raw FACsPbI_3_ and FAPbI_3_ films were simply fabricated by one-step spin-coating at 4000 rpm for 30 s and then heated at 140 °C for 20 min.

NH_3_-FACsPbI_3_ film: After spin-coating, the raw FACsPbI_3_ film was annealed at 140 °C for 5 min, and then transferred into a homemade chamber with a temperature from 20 to −15 °C. N_2_ gas was used to remove the moisture-laden air in the chamber. Subsequently, NH_3_ gas was quickly introduced into the chamber and maintained for ∼5 s. Then the NH_3_ gas is removed from the chamber and the film was further annealed at 140 °C for 20 min. For NH_3_-FAPbI_3_ film, the film treatment process is the same as above, and the temperature of the homemade chamber is −15 °C for NH_3_ post-healing process.

For anti-FACsPbI_3_, anti-FMCsPbI_3_, and anti-MAPbI_3_ films, the perovskite precursors were prepared in DMF: DMSO (3:7, 1.40 M) by stoichiometric ratios. The perovskite solution was spin-coated in a two-step at 1000 rpm and 4000 rpm for 10 s and 30 s, respectively. During the second step, 300 μl anisole was drop-casted quickly at the tenth second of the second step. The anti-MAPbI_3_ perovskite film was then heated at 100 °C for 20 min, and the other films were heated at 140 °C for 20 min.

The α-FAPbI_3_, δ-FAPbI_3_, MFAPbI_3_, and DMFAPbI_3_ films were fabricated from their DMF solution with a concentration of 1.40 M using chlorobenzene as the antisolvent. The α-FAPbI_3_, MFAPbI_3_, and DMFAPbI_3_ films were obtained after heating treatment at 140 °C for 20 min. The δ-FAPbI_3_ film was achieved by exposing α-FAPbI_3_ film in the air (RH > 50%) until fully transformed into yellow. The MA-FAPbI_3_ film was prepared by the MA^0^ gas post-healing the α-FAPbI_3_ film and then heated at 140 °C for 20 min.

The annealing process of all thin films is in ambient air conditions (30–40% humidity).

### Device fabrication

The methods described are similar to that reference 44. Fluorine doped tin oxide (FTO)-coated glass (2.20 mm, 7Ω sq^−1^) was used as the substrate for the devices. The compact TiO_2_ layer (∼10 nm) was deposited by atomic layer deposition (ALD) for 200 cycles and annealed at 500 °C for 30 min in ambient air. For ALD TiO_2_ deposition, titanium (IV) isopropoxide (TTIP) and H_2_O as Ti and O sources, respectively. The TTIP precursor was held at 75 °C. Pulse/exposure/purge times of 1 s/8 s/25 s were used for the TTIP and 0.1 s/8 s/25 s for H_2_O precursor, and the deposition temperature was set to 120 °C. On top of the c-TiO_2_ layer, SnO_x_-Cl layer was deposited by spin-coating at a speed of 3000 rpm for 30 s from an aged SnCl_4_ aqueous solution (1:75 with deionized water by volume), followed by a sintering heat-treatment of 200 °C for 30 min in air and then transferred to the glove box for device fabrication. The perovskite layers were fabricated as described above. For the surface passivation, 1 mg ml^−1^ of Phenyltrimethylammonium tribromide (PTAB) solution in isopropanol was spin-coated on these perovskite films at 4000 rpm for 30 s.

The spiro-OMeTAD chlorobenzene solution (72.3 mg ml^−1^) with 28.8 μl 4-tert-butylpyridine (96%, Aldrich-Sigma) and 17.5 μl lithium bis(trifluoro-methanesulfonyl) imide (Li-TSFI, Aldrich-Sigma) solution (520 mg Li-TSFI (98%) in 1 ml acetonitrile (99.8%, Aldrich-Sigma)) was spin-coated on top of the perovskite film at 3000 rpm for 30 s. The devices were put into a dry-air box (RH < 3%) for 12 h. Finally, 80 nm thick Au electrode was thermally evaporated.

For fabricating modules, P1 etching process was pre-patterned on FTO glass (5 cm × 5 cm) with a 1064 nm fiber laser (Han’s laser). The laser power ratio, laser duty cycle, and laser frequency were 30%, 5%, and 50 kHz, respectively. Then, patterned FTO substrates were cleaned and treated by UV Ozone Cleaner (Ossila) for 15 min. The TiO_2_/SnO_2_, PTAB, and Spiro-OMeTAD layers were prepared with the same procedure as presented above. The large-size raw FACsPbI_3_ perovskite film was prepared through the doctor-blading method (provided by Suzhou GCL Nano Co. Ltd.). The NH_3_ post-healing strategy was carried out on it to form a large-scale NH_3_-FACsPbI_3_ film. For P2 etching process, the laser used was a 532 nm laser with a laser power ratio of 65%, a laser duty cycle of 5%, and a laser frequency of 100 kHz. 80 nm thick Au electrodes were thermally evaporated under vacuum to complete the modules fabrication. Finally, P3 etching used the same laser with P2 with a laser power ratio of 50%, a laser duty cycle of 5%, and a laser frequency of 100 kHz. P4 is an etching procedure for cleaning the edge of the modules, the laser used in P4 is the same with P1 with a laser power ratio of 40%, the laser duty cycle of 10%, and a laser frequency of 100 kHz.

### Perovskite film and device characterization

The characterization described are similar to that reference 44. XRD spectra were measured by Ultima IV of Rigaku with Cu Kα radiation (1.5406 Å). The UV-Vis absorbance spectra were measured by QE Pro (Ocean Optics). Top view, cross-section SEM images were obtained with a field-emission SEM (S-4800, Hitachi). Steady PL spectra were recorded on QE Pro excited at 460 nm. AFM measurements were performed in contact mode (5400, Agilent). EQE measurement was calculated using certified incident photon to current conversion efficiency equipment from Enlitech (QE-R). Time-resolved photoluminescence (TRPL) experiments were performed by Steady State and Transient State Fluorescence Spectrometer (Edinburgh FLS980). The testing conditions as the films were photoexcited at 483.6 nm pulse width ∼ 118.6 ps, 5 mW/pulse, and emission were collected on the surface side of the film (perovskite/ glass substrate).

*J-V* curves of the as-fabricated PSCs were measured using a SourceMeter (Keithley 2400) under simulated one-sun AM 1.5 G 100 mW cm^−2^ intensity (Oriel Sol3A Class AAA, Newport) with a scan rate of 200 mV/s (the voltage step is 20 mV with no delay time) from reverse and forward two scanning directions in air condition around 25 °C. The typical active area of PSCs is 0.09 cm^2^ defined by a metal mask. The area of the mask (0.08713 cm^2^) used for certification was certified by the National Institute of Metrology, China, No. CDjc2021-10891. The intensity of one-sun AM 1.5 G illumination was calibrated using a Si-reference cell certified by the National Renewable Energy Laboratory. SPO is measured by tracking the current under a fixed voltage which is decided by the voltage of maximum power point at the *J-V* curve. Here, the fixed voltage of NH_3_-FACsPbI_3_ device is 1.002 V.

For the stability tests, all PSCs were without encapsulation. For shelf-life stability, the devices were stored in a dark environment with a humidity of 10–30%, and the photovoltaic performance of PSCs was measured every ten days. The operational stability was performed using a stability setup (LC Auto-Test 24, Shenzhen Lancheng Technology Co., Ltd.), tested under continuous light illumination and maximum power point tracking (controlled and monitored to be 15 °C). The light source consisted of an array of white LEDs powered by a constant current. The LED type is MG-A200A-AE with an emission spectrum of 400–750 nm (Supplementary Fig. 40). Equivalent sun intensities were calibrated using a calibrated Si-reference cell. During aging, the device is connected with a 100 Ohm load resistance. The PSCs were masked and placed inside a sample holder purged with continuous N_2_ flow. *J-V* curves with reverse voltage scans were recorded every 12 h during the whole operational test.

### Reporting summary

Further information on research design is available in the Nature Research Reporting Summary linked to this article.

## Supplementary information

Supplementary Information

Peer Review File

Solar Cells Reporting Summary

## Data Availability

Data that support the findings of this study are available in Supplementary Data Files in the Supplementary Information section. Source data are provided with this paper.

## Source data

Source Data
