# Supplementary material for: Ammonia for post-healing of formamidinium-based Perovskite films
Source: Nat Commun. 2022 Jul 29;13:4417. doi: 10.1038/s41467-022-32047-z (PMC9338283; doi:10.1038/s41467-022-32047-z)
Supplement: Supplementary file 1 — Supplementary Information [file 41467_2022_32047_MOESM1_ESM.pdf]

Supporting Information

Ammonia for Post Healing of Formamidinium-based Perovskite Films

Zhipeng Li,[1,2] Xiao Wang,[1] Zaiwei Wang,[1] Zhipeng Shao,[1] Lianzheng Hao,[1,2] Yi Rao,[1,2] Chen Chen,[1] Dachang Liu,[1,2] Qiangqiang Zhao,[1,3] Xiuhong Sun,[1,2] Caiyun Gao,[1] Bingqian Zhang,[1] Xianzhao Wang,[1,2] Li Wang,[3,*] Guanglei Cui,[1,4,*] and Shuping Pang[1,*]

[1]Qingdao Institute of Bioenergy and Bioprocess Technology, Chinese Academy of Sciences, Qingdao 266101, P. R. China

[2]Center of Materials Science and Optoelectronics Engineering, University of Chinese Academy of Sciences, Beijing 100049, P. R. China

[3]College of Materials Science and Engineering, Qingdao University of Science and Technology, Qingdao, 266042, P. R. China

[4]School of Future Technology, University of Chinese Academy of Sciences, Beijing 100049, P. R. China

Corresponding Author

Li Wang - Email: liwang718@qust.edu.cn
Guanglei Cui - Email: cuigl@qibebt.ac.cn
Shuping Pang - China Email: pangsp@qibebt.ac.cn

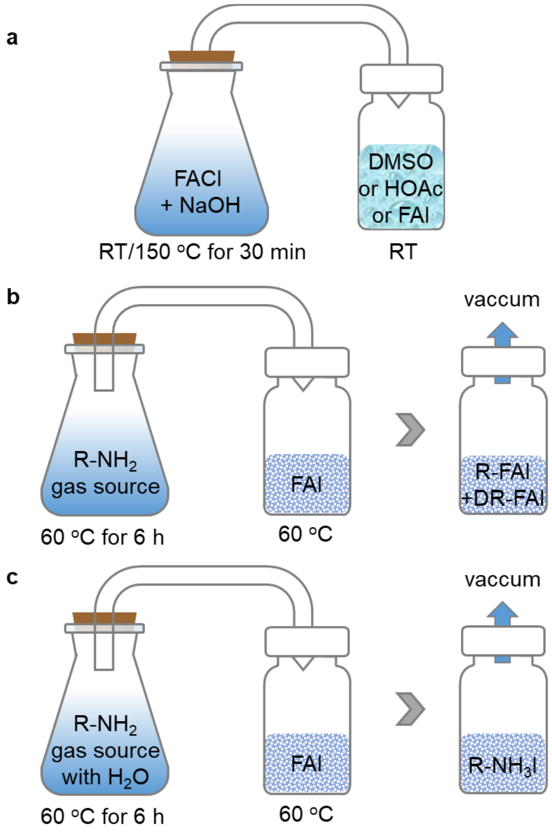

Supplementary Fig. 1 Schematic illustration of (a) the chemical reaction between FACl and NaOH powders with the gas collection by DMSO-d_6, HOAc, or FAI, (b, c) the chemical reactions between amine gases (R-NH_2) and FAI salt in (b) dry condition or (c) wet condition.

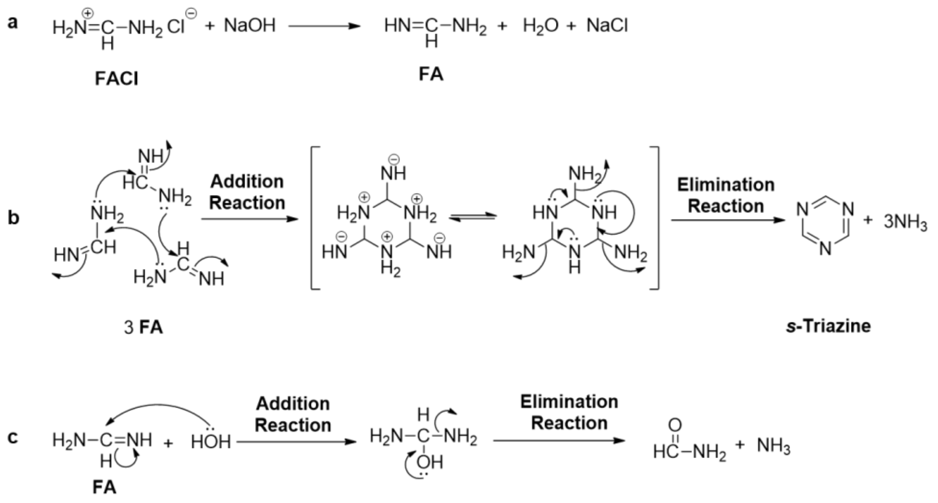

Supplementary Fig. 2 (a) The formation of FA^0 from the reaction of FACl and NaOH powders. (b) The reaction mechanism of transimination reaction among FA^0 molecules. (c) The decomposition of the FA^0 in the presence of H_2O.

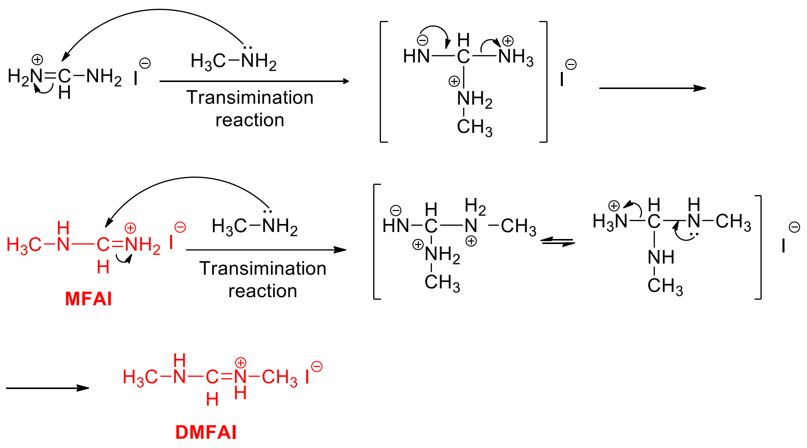

Supplementary Fig. 3 Reaction mechanism of transimination reactions between MA and FAI.

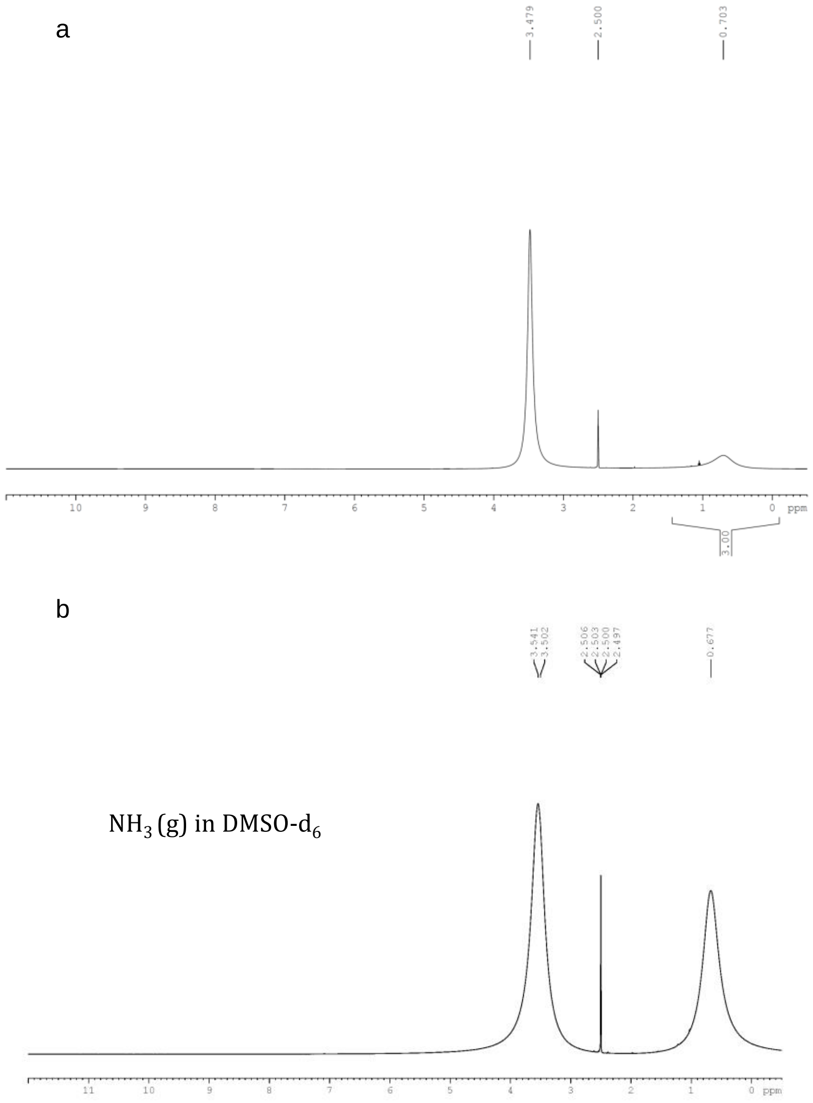

Supplementary Fig. 4 (a) The ^{1}H NMR spectrum of the reaction product between FACl and NaOH powders under room temperature directly absorbed by DMSO-d_6. (b) Commercial NH_3 gas directly absorbed by DMSO-d_6 under room temperature. The peaks were assigned as: NH_3, δ 0.70 for (a) and 0.68 for (b) (br, -N-H); HDO, δ 3.48 for (a) and 3.54 for (b) (br, HDO); DMSO-d_6 residual peak, δ 2.50.

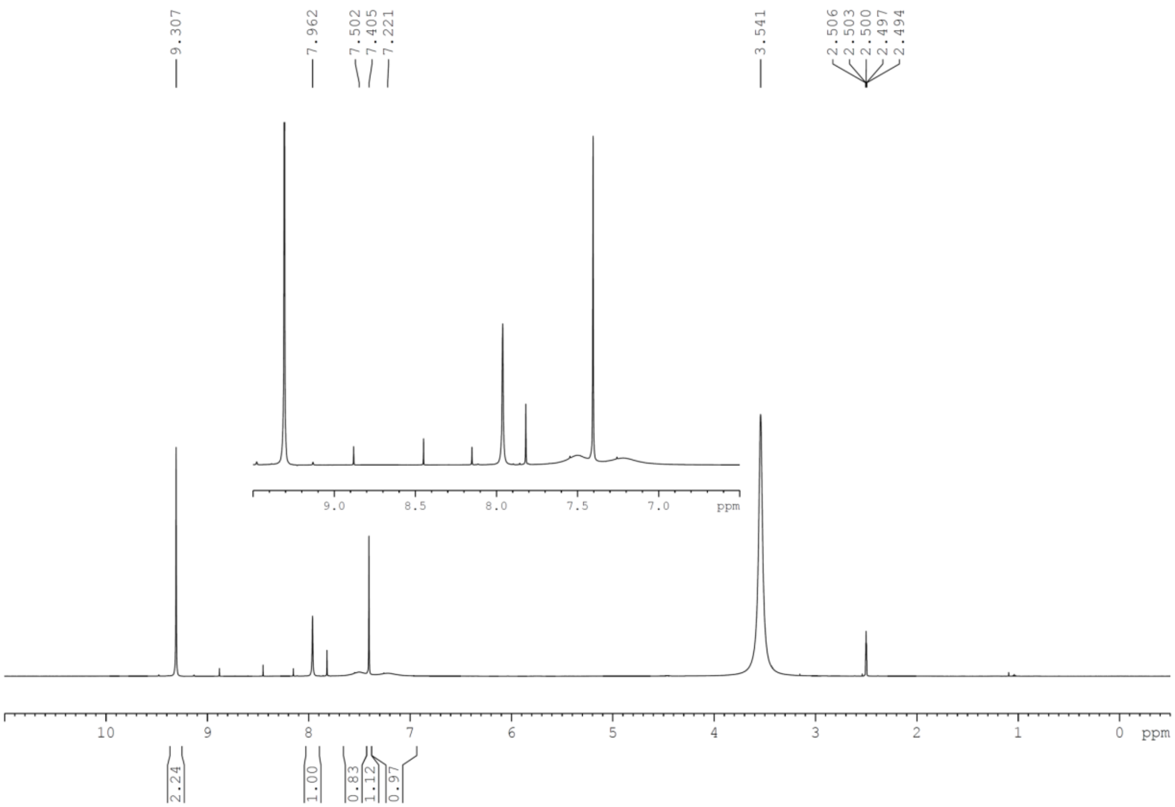

Supplementary Fig. 5 The ^{1}H NMR spectrum of reaction products between FACl and NaOH powders under 150 ˚C directly absorbed by DMSO-d_6. The peaks were assigned as: s-triazine, δ 9.30(s, -N=C<u>H</u>); formamide, δ 7.96(s, <u>H</u>-C=O), 7.50(br, -N-<u>H</u>), 7.22(br, -N-<u>H</u>); FA, δ 7.41(s, N-<u>H</u>C=N); HDO, δ 3.54 (br, <u>H</u>DO); DMSO-d_6 residual peak, δ 2.50.

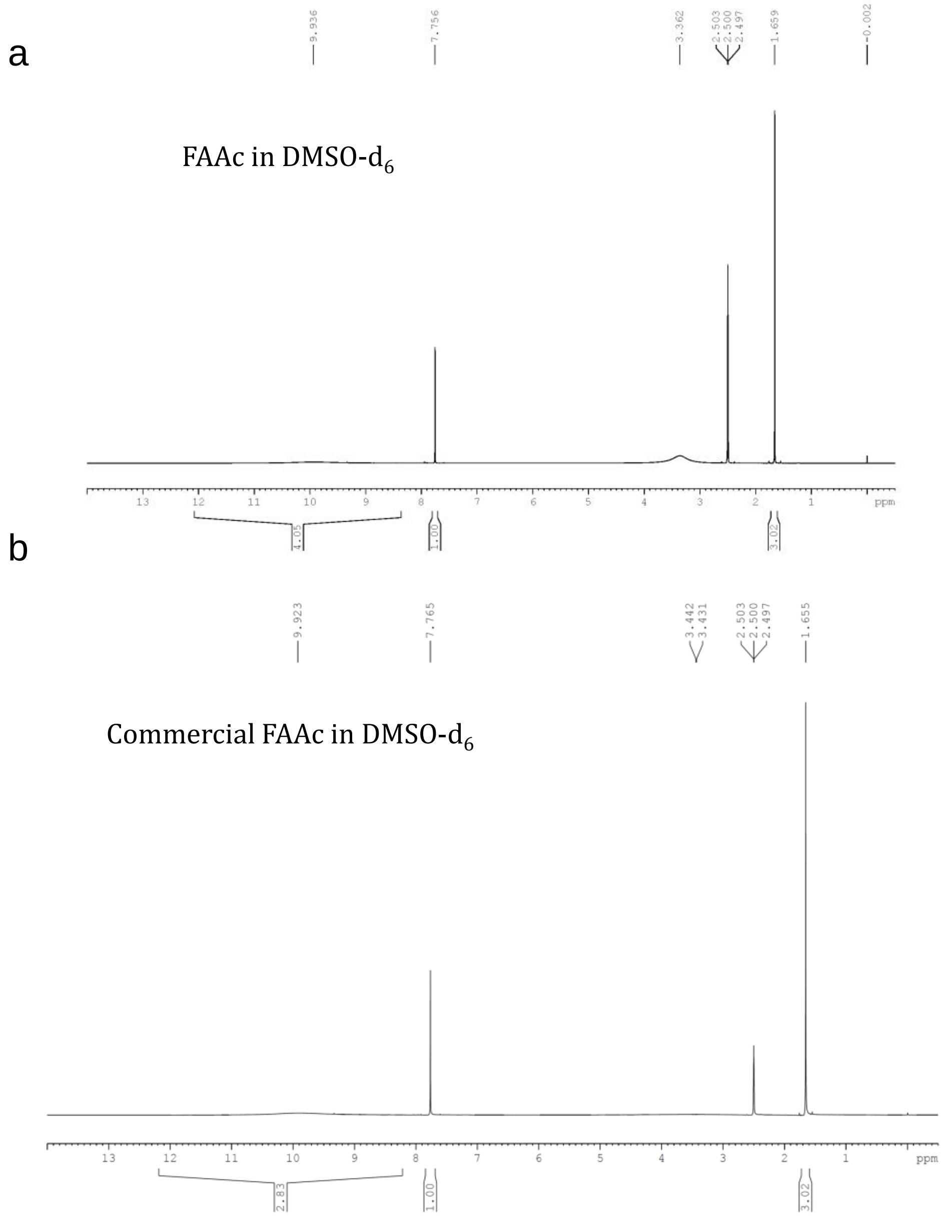

Supplementary Fig. 6 The ^{1}H NMR spectrum (DMSO-d_6) of reaction product between FACl and NaOH powders under 150 ˚C absorbed by HOAc (a) and the commercial FAAc for comparison (b). The peaks were assigned as: FAAc, δ 9.94 for (a) and 9.92 for (b)(br, N-H), 7.76(s, N-HC=N), 1.66(s, -CH_3); HDO, δ 3.36 for (a)(br, HDO); DMSO-d_6 residual peak, δ 2.50.

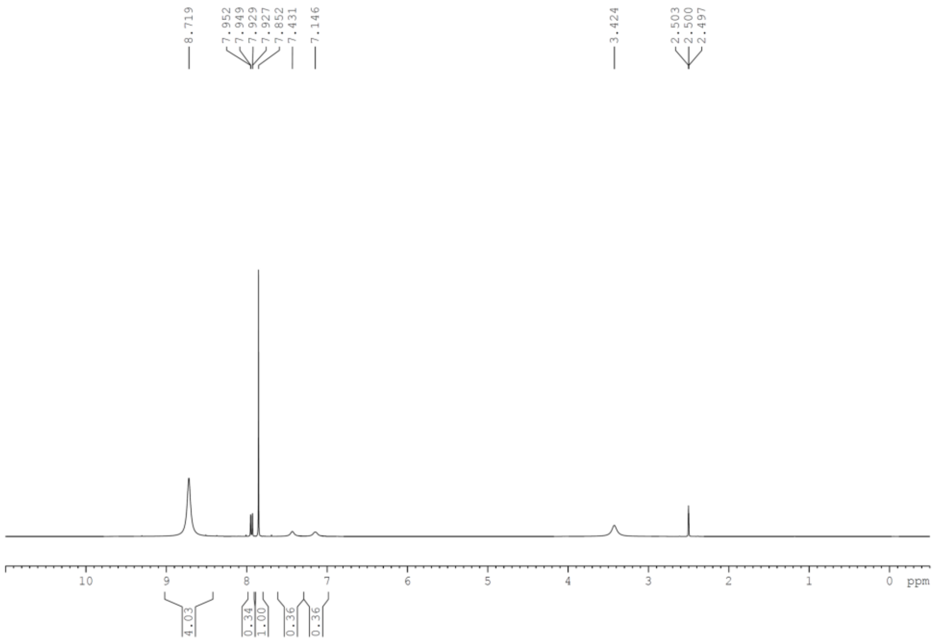

Supplementary Fig. 7 The ^{1}H NMR spectrum (DMSO-d_6) of reaction products between FAI powder and FA^0. The peaks were assigned as: FAI, δ 8.72(br, N-<u>H</u>), 7.85(s, N-<u>H</u>C=N); formamide, δ 7.95(dd, J_1 = 1.4 Hz, J_2 = 13.5 Hz, <u>H</u>-C=O), 7.43(br, -N-<u>H</u>), 7.15(br, -N-<u>H</u>); HDO, δ 3.54 (br, <u>H</u>DO); DMSO-d_6 residual peak, δ 2.50.

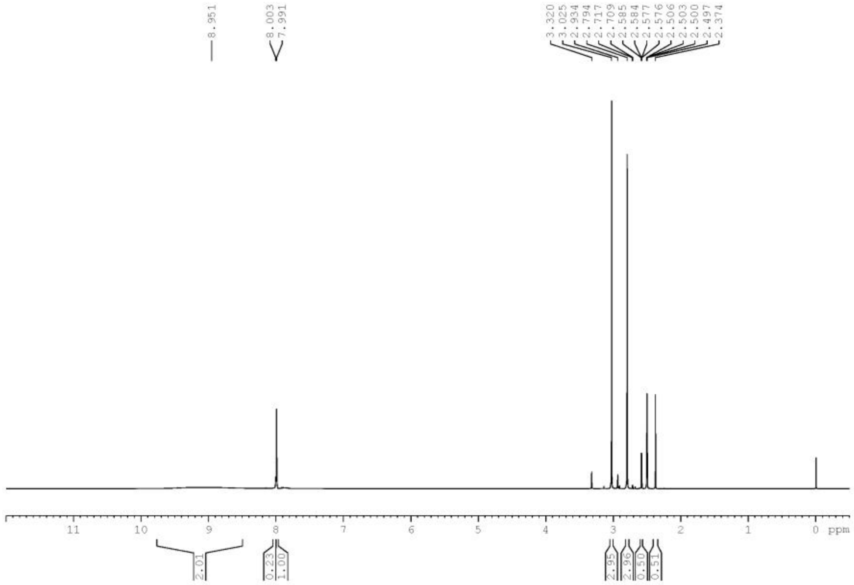

Supplementary Fig. 8 The ^{1}H NMR spectrum (DMSO-d_6) of the reaction product between FAI powder and MA^0. The peaks were assigned as: DMFAI, δ 8.95(br, N-<u>H</u>), 7.99(s, N-<u>H</u>C=N), 3.03(s, C=N-C<u>H</u>$_3$), 2.79(s, C-N-C<u>H</u>$_3$); MFA, δ 8.00(br, N-<u>H</u>), 2.58(dd, J_1 = 0.54 Hz, J_2 = 4.8 Hz, N-C<u>H</u>$_3$); MAI, δ 2.37(s, N-C<u>H</u>$_3$); HDO, δ 3.32 (br, <u>H</u>DO); DMSO-d_6 residual peak, δ 2.50.

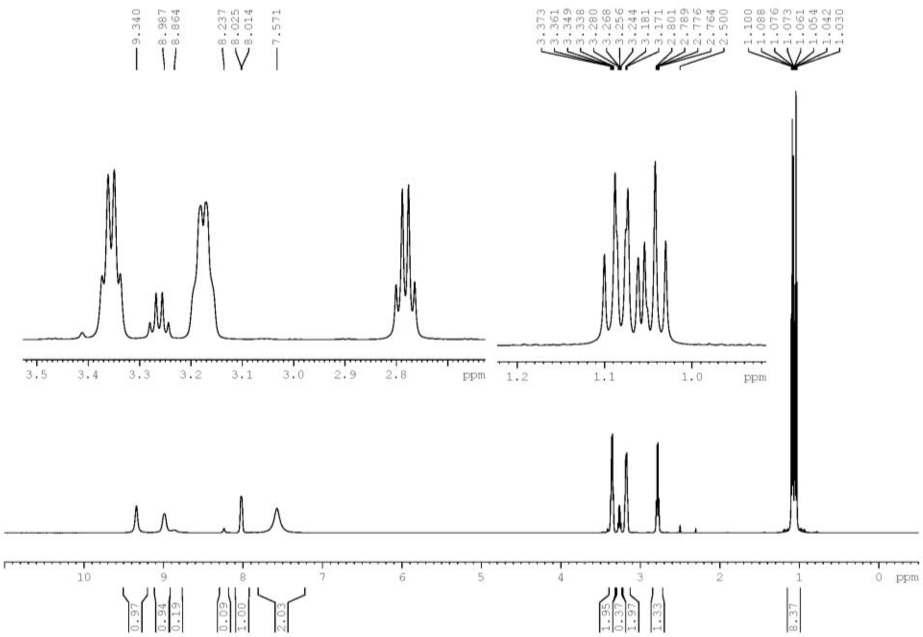

Supplementary Fig. 9 The ^{1}H NMR spectrum (DMSO-d_6) of reaction products between FAI powder and EA^0. The peaks were assigned as: DEFAI, δ 9.34(br, N-H), 8.99(br, N-H), 8.02(d, *J* = 6.72 Hz, N-HC=N), 3.35(q, *J* = 7.06 Hz, C=N-CH_2), 3.18(q, *J* = 6.40 Hz, C-N-CH_2), 1.06(m, N-CH_2-CH_3); DEFA, δ 8.86(br, N-H), 8.24(s, N-HC=N), 3.26(q, *J* = 7.20 Hz, C-N-CH_2), 1.06(m, N-CH_2-CH_3); EAI, δ 7.57(br, N-H), 2.78(q, *J* = 7.24 Hz, C-N-CH_2), 1.06 (m, N-CH_2-CH_3); DMSO-d_6 residual peak, δ 2.50.

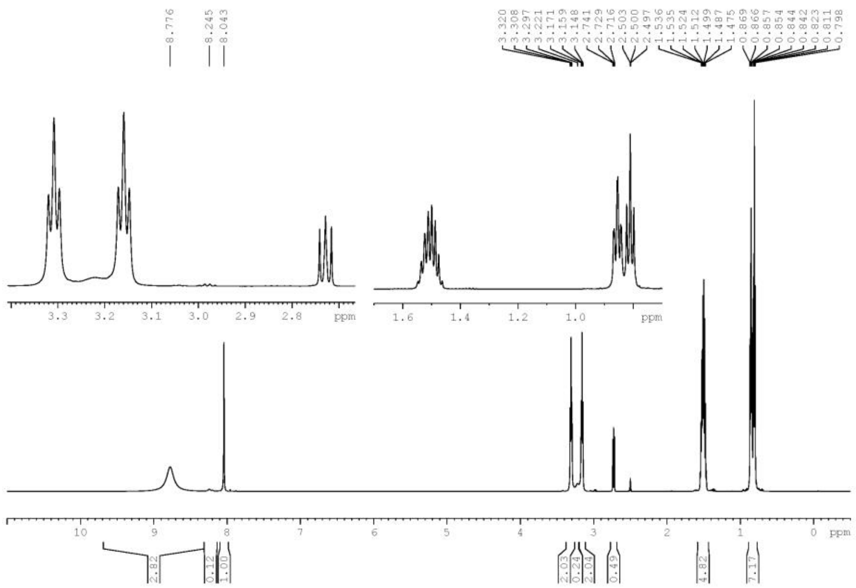

Supplementary Fig. 10 The ^{1}H NMR spectrum (DMSO-d_6) of reaction products between FAI powder and PA^0. The peaks were assigned as: DPFAI, δ 8.78(br, N-$\underline{H}$), 8.02(s, N-$\underline{H}$C=N), 3.30(t, *J* = 6.96 Hz, C=N-C$\underline{H_2}$), 3.16(t, *J* = 7.05 Hz, C-N-C$\underline{H_2}$), 1.50(m, N-CH_2-C$\underline{H_2}$), 0.85(m, N-CH_2-CH_2-C$\underline{H_3}$); DPFA, δ 8.25(br, N-$\underline{H}$), 8.20(s, N-$\underline{H}$C=N), 3.22(m, C-N-C$\underline{H_2}$), 1.50(m, N-CH_2-C$\underline{H_2}$), 0.85(m, N-CH_2-CH_2-C$\underline{H_3}$); PAI, δ 8.78(br, N-$\underline{H}$), 2.73(t, *J* = 7.50 Hz, C-N-C$\underline{H_2}$), 1.50(m, N-CH_2-C$\underline{H_2}$), 0.85(m, N-CH_2-CH_2-C$\underline{H_3}$); DMSO-d_6 residual peak, δ 2.50.

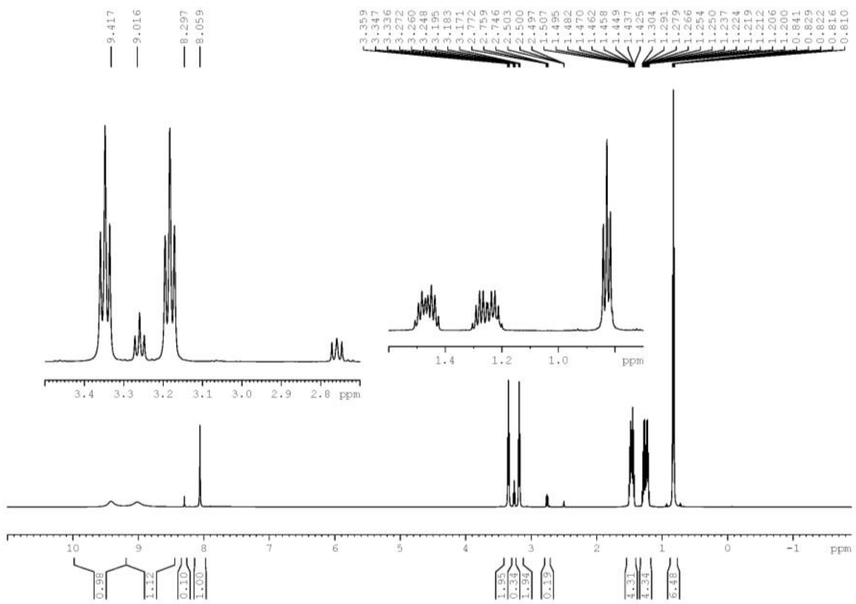

Supplementary Fig. 11 The ^{1}H NMR spectrum (DMSO-d_6) of the reaction product between FAI powder and BA^0. The peaks were assigned as: DBFAI, δ 9.42(br, N-H), 9.02(br, N-H), 8.06(s, N-HC=N), 3.35(t, *J* = 7.11 Hz, C=N-CH_2), 3.18(t, *J* = 7.14 Hz, C-N-CH_2), 1.47(m, N-CH_2-CH_2), 1.25(m, N-CH_2-CH_2-CH_2), 0.82(m, N-CH_2-CH_2-CH_2-CH_3); DBFA, δ 9.02(br, N-H), 8.30(s, N-HC=N), 3.26(t, *J* = 7.05 Hz, C-N-CH_2), 1.25(m, N-CH_2-CH_2-CH_2), 0.82(m, N-CH_2-CH_2-CH_2-CH_3); BAI, δ 2.76(t, *J* = 7.59 Hz, C-N-CH_2), 1.25(m, N-CH_2-CH_2-CH_2), 0.82(m, N-CH_2-CH_2-CH_2-CH_3); DMSO-d_6 residual peak, δ 2.50.

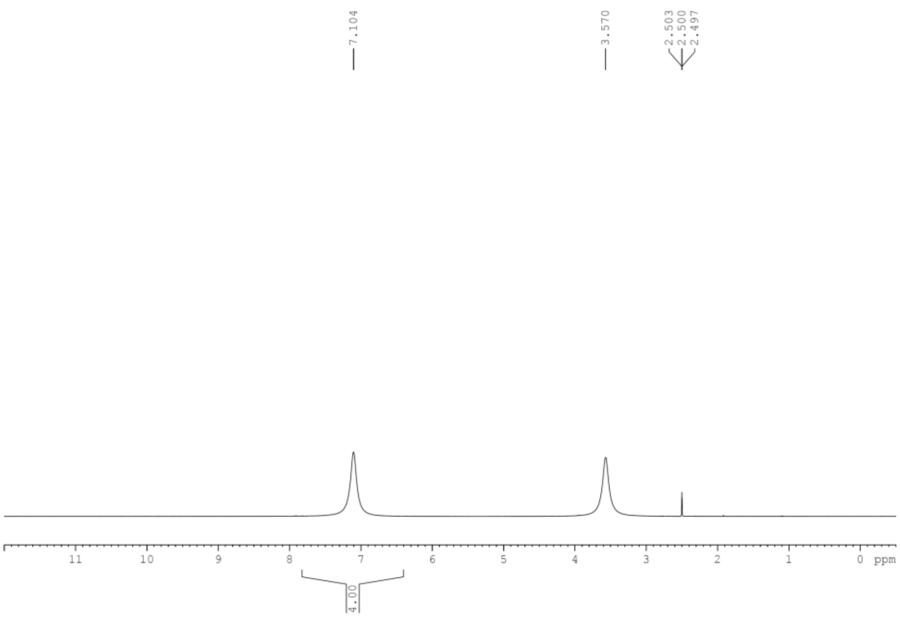

Supplementary Fig. 12 The ^{1}H NMR spectrum (DMSO-d_6) of reaction product between FAI powder and NH_3 with the presence of H_2O. The peaks were assigned as: NH_4I, δ 7.10(s, N-<u>H</u>); HDO, 3.57(s, <u>H</u>DO); DMSO-d_6 residual peak, δ 2.50.

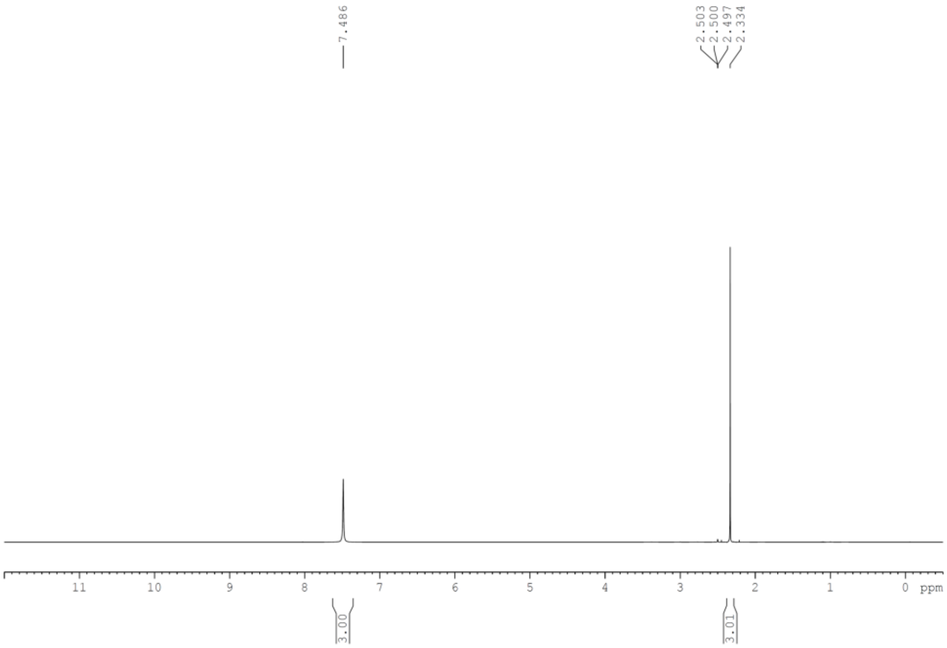

Supplementary Fig. 13 The ^{1}H NMR spectrum (DMSO-d_6) of reaction product between FAI powder and MA^0 with the presence of H_2O. The peaks were assigned as: MAI, δ 7.49(s, N-<u>H</u>), 2.33(s, N-C<u>H3</u>); DMSO-d_6 residual peak, δ 2.50.

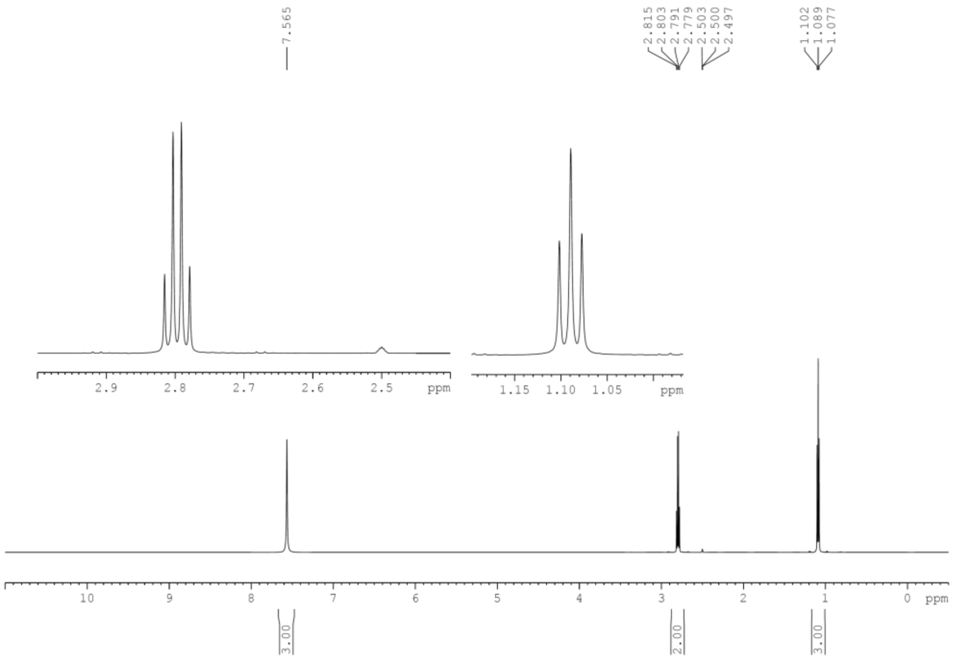

Supplementary Fig. 14 The ^{1}H NMR spectrum (DMSO-d$_6$) of reaction product between FAI powder and EA^0 with the presence of H_2O. The peaks were assigned as: EAI, δ 7.57(s, N-H); 2.80(q, *J* = 7.28 Hz, N-CH_2), 1.09(t, *J* = 7.32 Hz, N-CH$_2$-CH_3); DMSO-d$_6$ residual peak, δ 2.50.

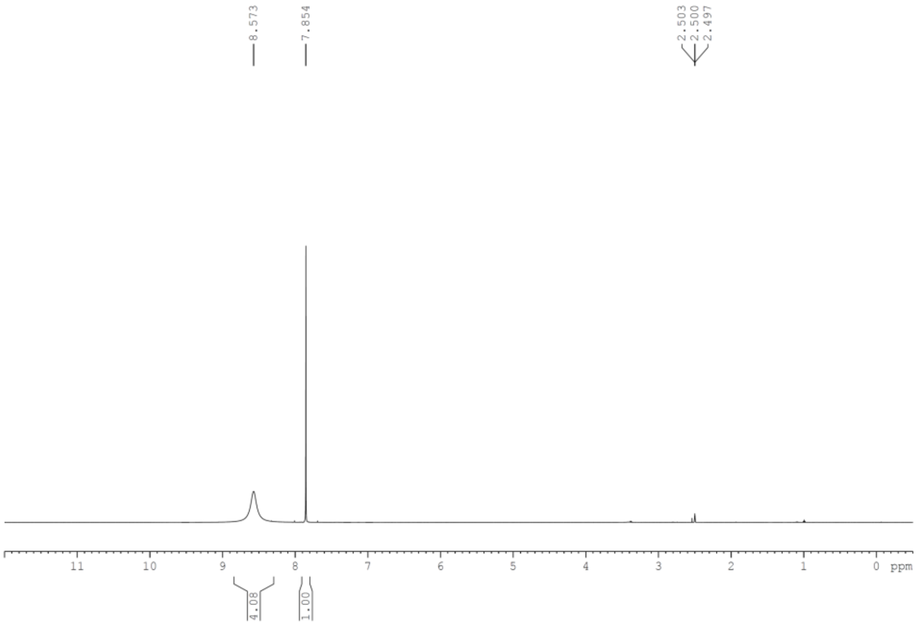

Supplementary Fig. 15 The ^{1}H NMR spectrum (DMSO-d$_6$) of reaction product between FAI powder and H_2O gas. The peaks were assigned as: FAI, δ 8.57(s, N-H), 7.85(s, N-CH_2), N-HC=N); DMSO-d$_6$ residual peak, δ 2.50.

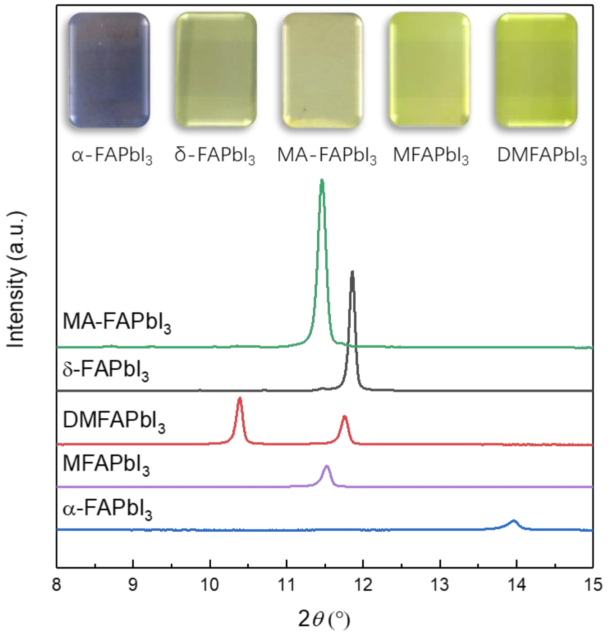

Supplementary Fig. 16 The XRD patterns of α-$FAPbI_3$, δ-$FAPbI_3$, $MFAPbI_3$, $DMFAPbI_3$ and MA-$FAPbI_3$ films (MA-$FAPbI_3$ refers to the MA^0 post healing α-$FAPbI_3$ film).

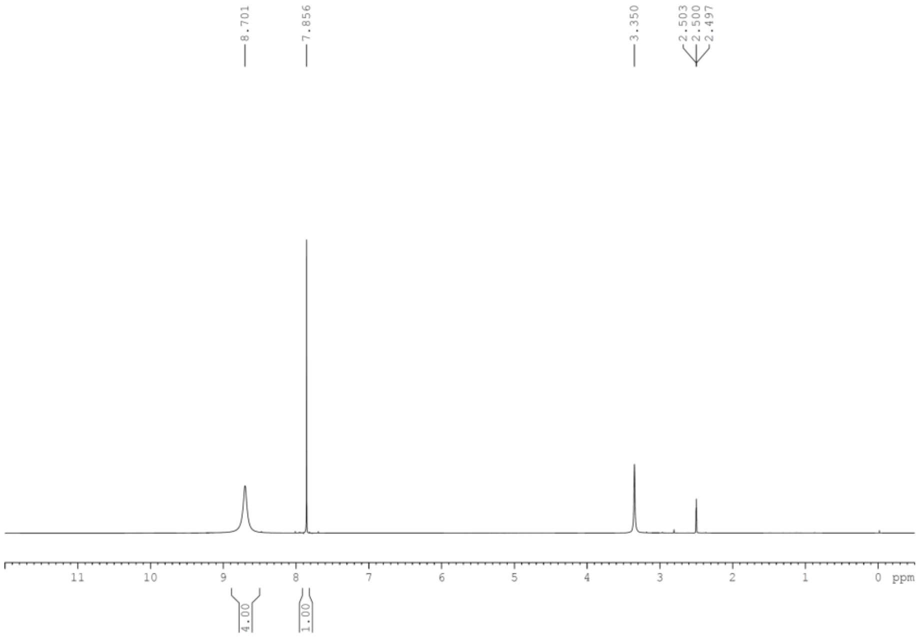

Supplementary Fig. 17 The ^1H NMR spectrum (DMSO-d_6) of reaction product between FAI powder and NH_3. The peaks were assigned as: FAI, δ 8.70(s, N-H), 7.86(s, N-CH_2), N-HC=N); HDO, 3.57(s, HDO); DMSO-d_6 residual peak, δ 2.50.

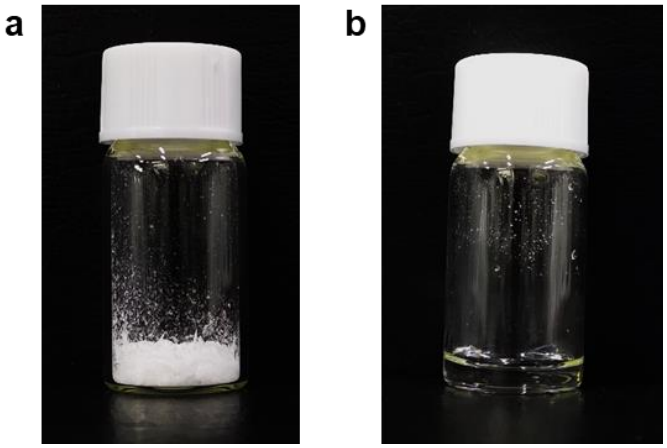

Supplementary Fig. 18 Photographs of (a) FAI powder, (b) FAI·xNH_3.

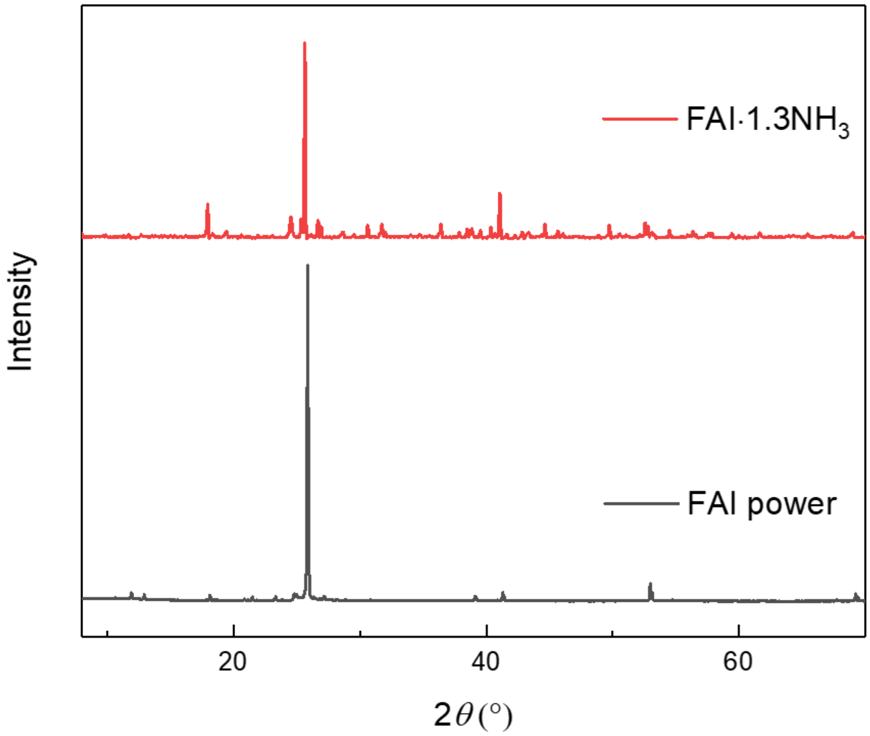

Supplementary Fig. 19 The XRD patterns of FAI·1.3NH_3 and FAI powder.

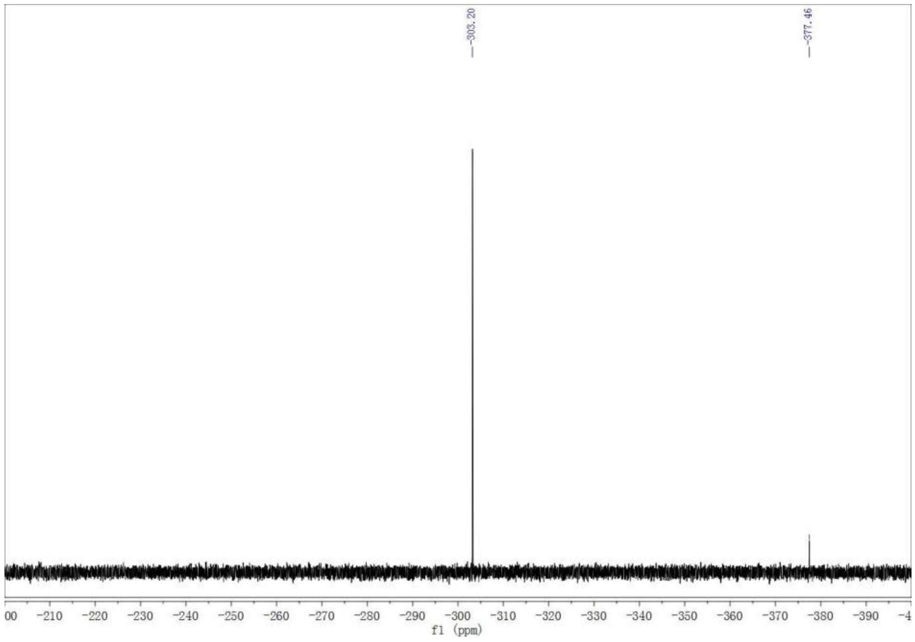

Supplementary Fig. 20 The solution ^{15}N NMR spectrum (DMSO-d_6) of $^{15}NH_3$. The peak was assigned as: δ -377.46 ppm, ^{15}N signal in $^{15}NH_3$. The ^{15}N chemical shifts were determined from 1 M urea in DMSO (-303.2 ppm, 10% ^{15}N labelled) as external standard reference.

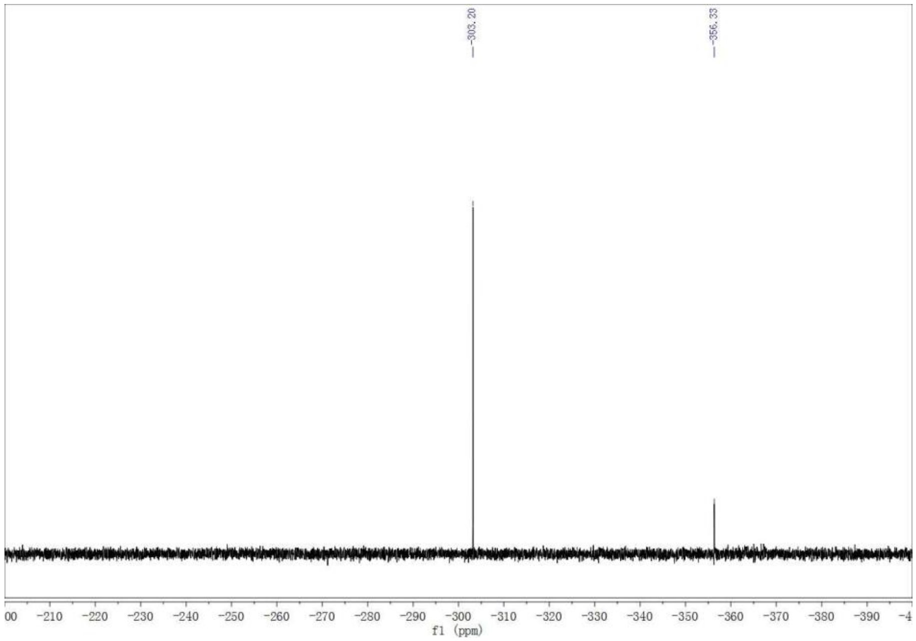

Supplementary Fig. 21 The solution ^{15}N NMR spectrum (DMSO-d_6) of FAI(^{15}N). The peak was assigned as: δ -356.33 ppm, ^{15}N signal in FAI (^{15}N). The ^{15}N chemical shifts were determined from 1 M urea in DMSO (-303.2 ppm, 10% ^{15}N labelled) as external standard reference.

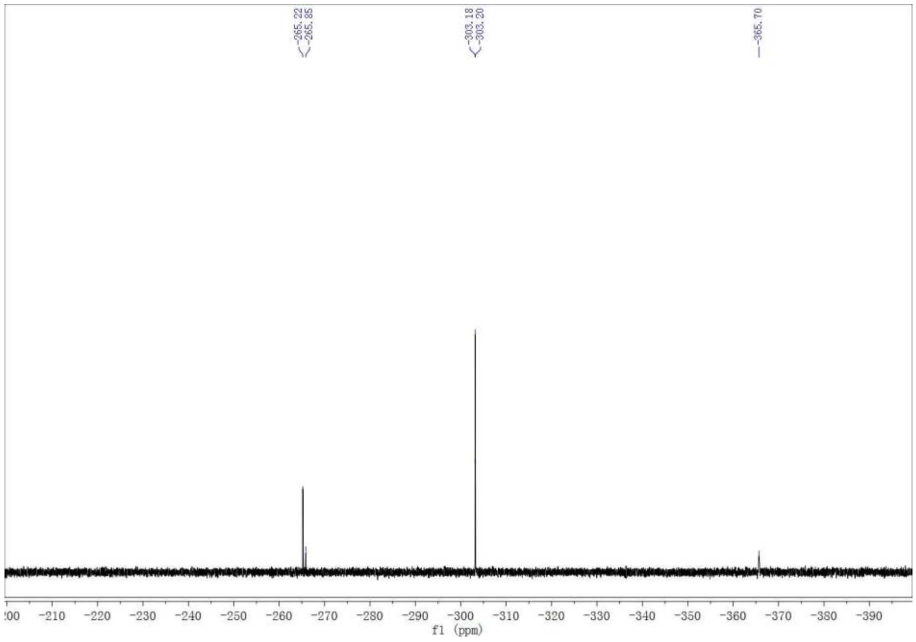

Supplementary Fig. 22 The solution ^{15}N NMR spectrum (DMSO-d_6) of FAI·x$^{15}NH_3$. The peak was assigned as: δ -265.22 ppm, ^{15}N signal in FAI hydrogen bonded with $^{15}NH_3$. The ^{15}N chemical shifts were determined from 1 M urea in DMSO (-303.2 ppm, 10% ^{15}N labelled) as external standard reference.

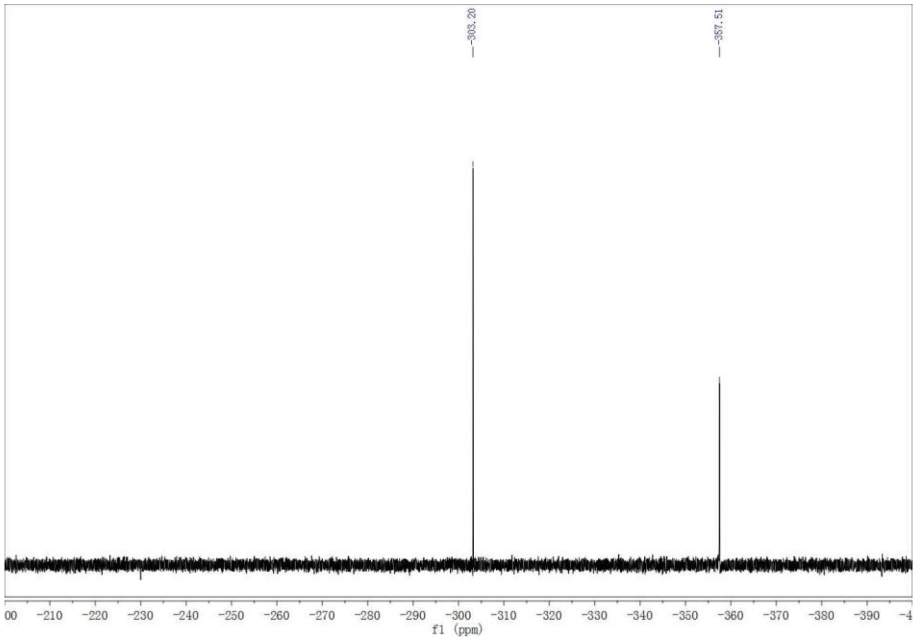

Supplementary Fig. 23 The solution ^{15}N NMR spectrum (DMSO-d_6) of PbI_2·x$^{15}NH_3$. The peak was assigned as: δ -357.51 ppm, ^{15}N signal of $^{15}NH_3$ coordinated with Pb(II). The ^{15}N chemical shifts were determined from 1 M urea in DMSO (-303.2 ppm, 10% ^{15}N labelled) as external standard reference.

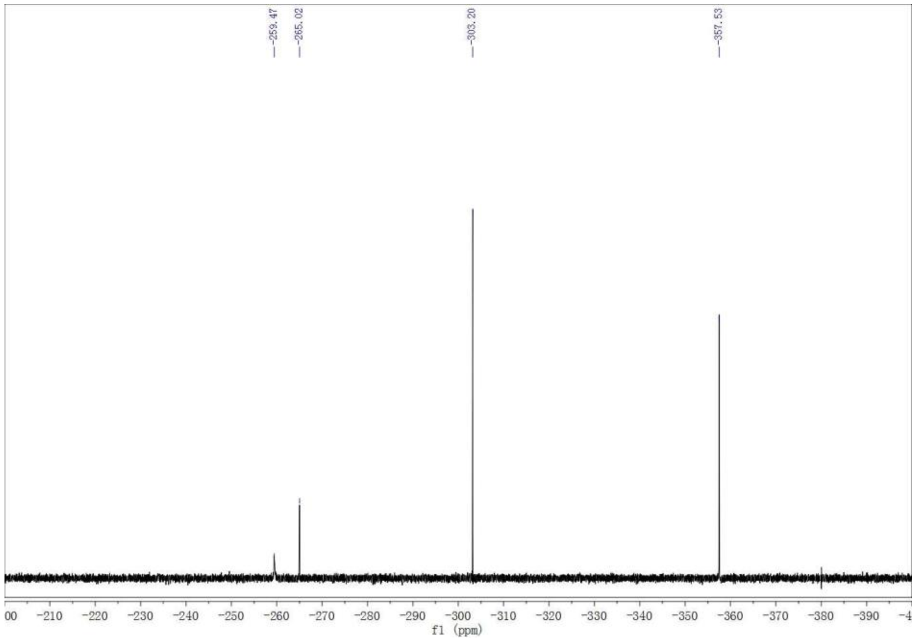

Supplementary Fig. 24 The solution ^{15}N NMR spectrum (DMSO-d_6) of $FAPbI_3{\cdot}x^{15}NH_3$. The peaks were assigned as: δ -259.47 ppm, ^{15}N signal of $^{15}NH_3$, which have both coordination interaction and hydrogen bond interaction; -265.02 ppm, ^{15}N signal in FAI hydrogen bonded with $^{15}NH_3$; -357.53 ppm, ^{15}N signal of $^{15}NH_3$ coordinated with Pb(II). The ^{15}N chemical shifts were determined from 1 M urea in DMSO (-303.2 ppm, 10% ^{15}N labelled) as external standard reference.

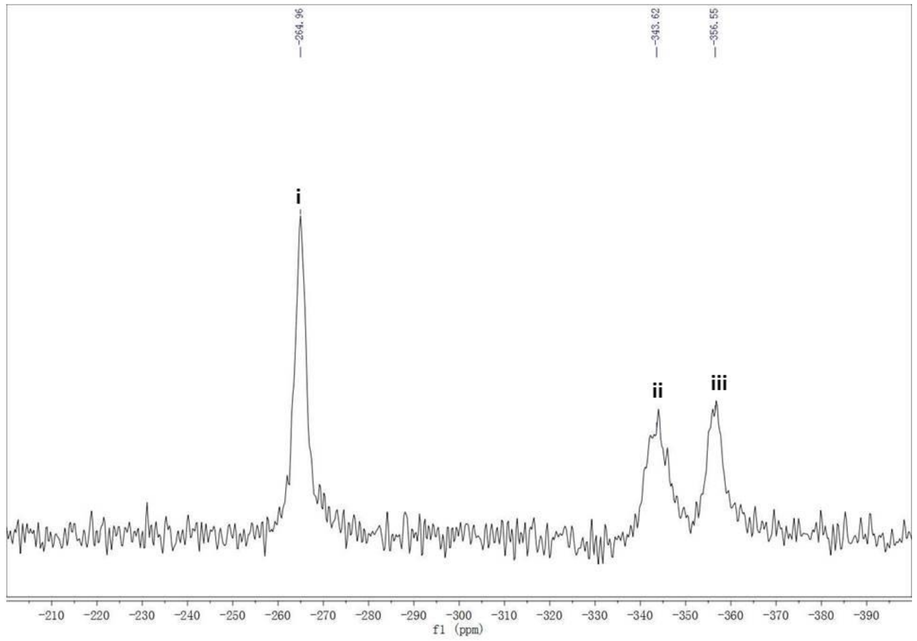

Supplementary Fig. 25 The solid-state ^{15}N NMR spectrum of $FAPbI_3{\cdot}x^{15}NH_3$. Signals: i, ^{15}N signal in FAI(^{15}N) hydrogen-bonded with NH_3; ii, ^{15}N signal in $^{15}NH_3$ coordinated with PbI_2; iii, ^{15}N signal in FAI(^{15}N).

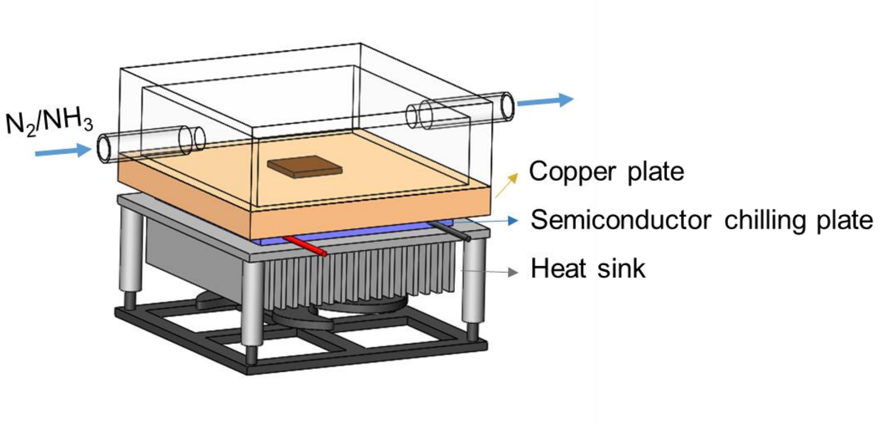

Supplementary Fig. 26 Schematic drawing of the home-made chamber with a semiconductor chilling plate to control the temperature.

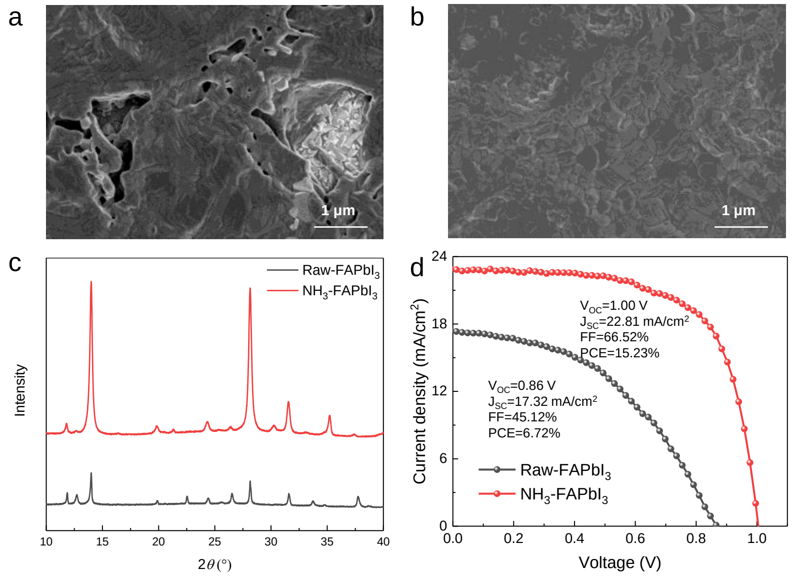

Supplementary Fig. 27 SEM images (a,b), XRD patterns (c) of Raw-$FAPbI_3$ and NH_3-$FAPbI_3$ films. (d) *J*-*V* curves of Raw- and NH_3-$FAPbI_3$ devices

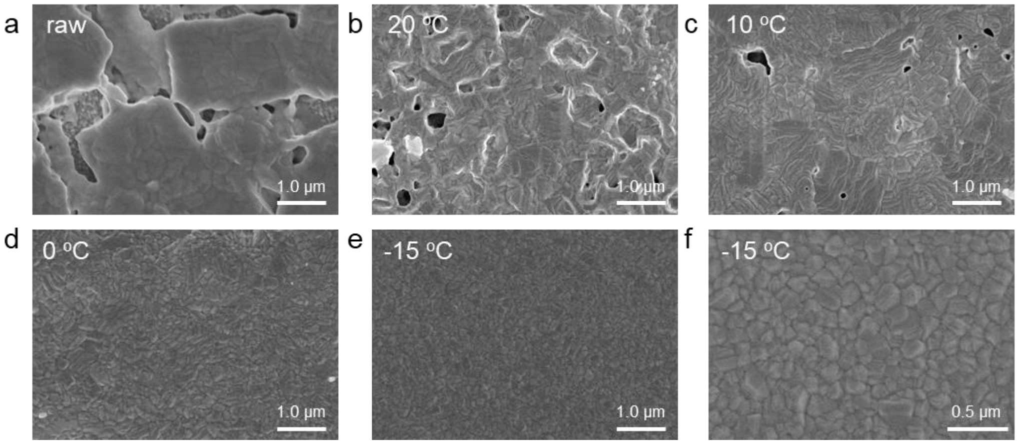

Supplementary Fig. 28 SEM images of the raw $FACsPbI_3$ perovskite film and the NH_3-$FACsPbI_3$ perovskite films fabricated at different temperatures (20 ˚C, 10 ˚C, 0 ˚C, -15 ˚C) of the NH_3 post healing process.

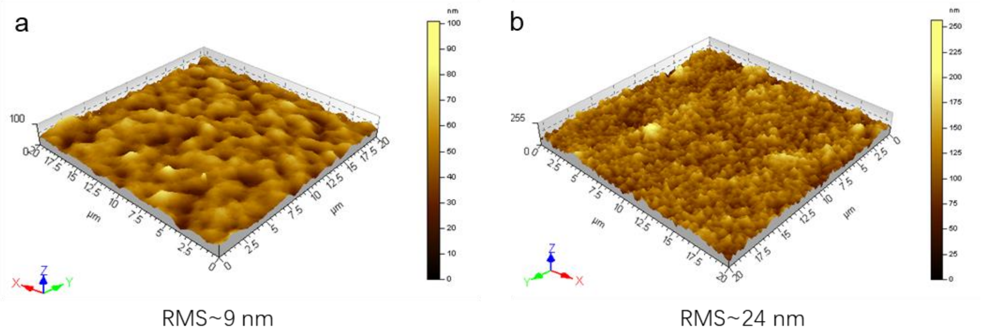

Supplementary Fig. 29 AFM images of top surfaces of (a) NH_3-$FACsPbI_3$ and (b) anti-$FACsPbI_3$ perovskite films.

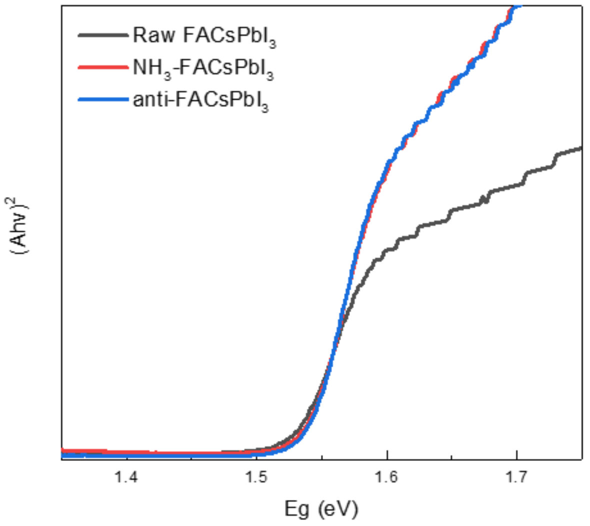

Supplementary Fig. 30 Tauc-plot of the Raw-$FACsPbI_3$, NH_3-$FACsPbI_3$ and anti-$FACsPbI_3$ films.

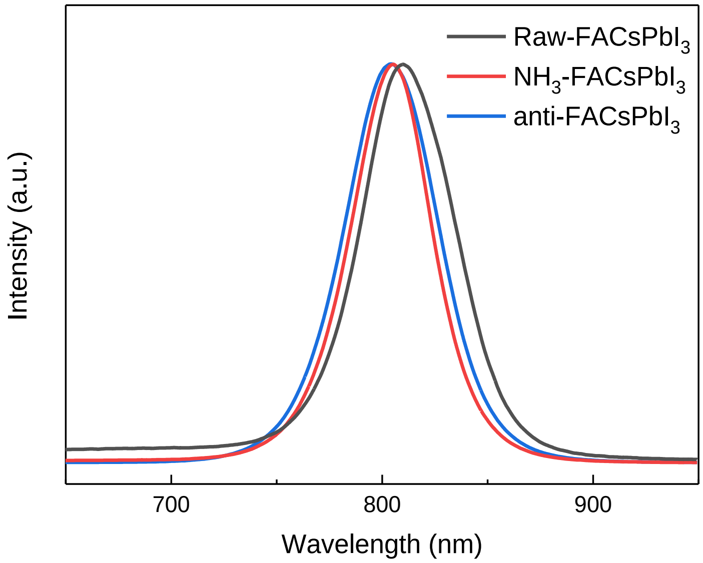

Supplementary Fig. 31 PL spectra of the Raw-$FACsPbI_3$, NH_3-$FACsPbI_3$ and anti-$FACsPbI_3$ films.

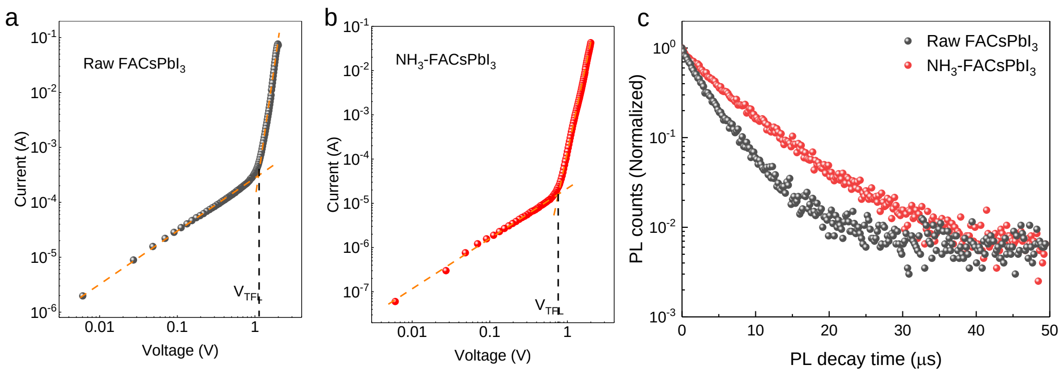

Supplementary Fig. 32 (a, b) J-V characteristics derived from the SCLC measurements with a structure of FTO/SnO_2/perovskite/PCBM/Au and (c) Time-resolved photoluminescence decay curves of the Raw and NH_3-$FACsPbI_3$ films.

The preparation details of thin film samples are as follows,

Raw $FACsPbI_3$ films: The perovskite precursor is prepared by mixing PbI_2: FAI: CsI (1:0.9:0.1 molar ratio) in DMF solvent (1.40 M). The Raw $FACsPbI_3$ films are simply fabricated by one-step spin coating at 4000 rpm for 30 s, and then heated at 140 °C for 20 min in ambient air conditions (30–40% humidity).

NH_3-$FACsPbI_3$ film: After spin coating, the raw $FACsPbI_3$ film is annealed at 140 °C for 5 min in ambient air conditions (30–40% humidity), and then transferred into a home-made chamber with the temperature around -15 °C. N_2 gas is used to remove the moisture-laden air in the chamber. Subsequently, NH_3 gas is quickly introduced into the chamber and maintained for ~5 s. Then the NH_3 gas is removed from the chamber and the film is further annealed at 140 °C for 20 min in ambient air conditions (30–40% humidity).

After annealing, some perovskite films are spin coated surface protective layer for the TRPL test, 50 mg ml^{-1} of polymethyl methacrylate (PMMA) solution in chlorobenzene was spin-coated on some films surface at 3000 rpm for 30 s and without further annealing process.

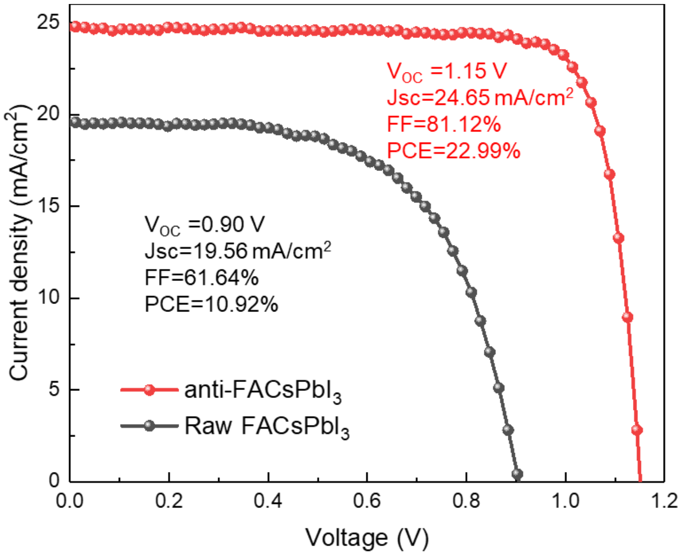

Supplementary Fig. 33 *J-V* curves of the Raw- and anti-$FACsPbI_3$ PSCs.

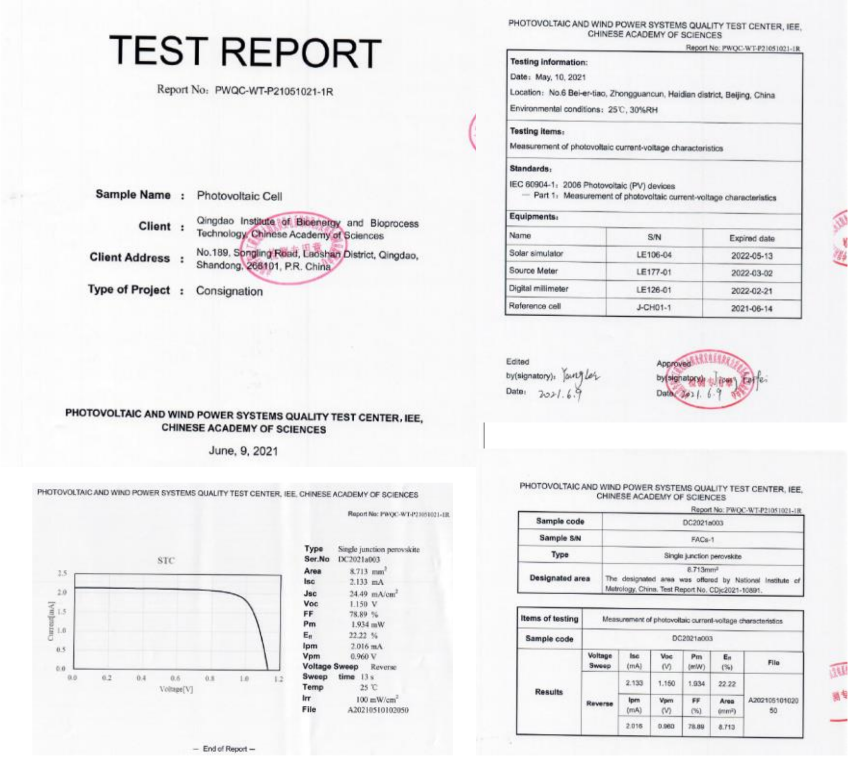

TEST REPORT

Report No: PWQC-WT-P21051021-1R

Sample Name : Photovoltaic Cell

Client : Qingdao Institute of Bioenergy and Bioprocess Technology, Chinese Academy of Sciences

Client Address : No.189, Songling Road, Laoshan District, Qingdao, Shandong, 266101, P.R. China

Type of Project : Consignation

PHOTOVOLTAIC AND WIND POWER SYSTEMS QUALITY TEST CENTER, IEE, CHINESE ACADEMY OF SCIENCES

June, 9, 2021

PHOTOVOLTAIC AND WIND POWER SYSTEMS QUALITY TEST CENTER, IEE, CHINESE ACADEMY OF SCIENCES

Report No: PWQC-WT-P21051021-1R

Testing information:
Date: May, 10, 2021
Location: No.6 Bei-er-tiao, Zhongguancun, Haidian district, Beijing, China
Environmental conditions: 25℃, 30%RH

Testing items:
Measurement of photovoltaic current-voltage characteristics

Standards:
IEC 60904-1: 2006 Photovoltaic (PV) devices
— Part 1: Measurement of photovoltaic current-voltage characteristics

Equipments:

Name	S/N	Expired date
Solar simulator	LE106-04	2022-05-13
Source Meter	LE177-01	2022-03-02
Digital millimeter	LE126-01	2022-02-21
Reference cell	J-CH01-1	2021-06-14

Edited by(signatory):
Date: 2021.6.9

Approved by(signatory):
Date: 2021. 6.9

PHOTOVOLTAIC AND WIND POWER SYSTEMS QUALITY TEST CENTER, IEE, CHINESE ACADEMY OF SCIENCES

Report No: PWQC-WT-P21051021-1R

STC

Current[mA]
Voltage[V]

Type	Single junction perovskite
Ser.No	DC2021a003
Area	8.713 mm^2
Isc	2.133 mA
Jsc	24.49 mA/cm^2
Voc	1.150 V
FF	78.89 %
Pm	1.934 mW
E_{ff}	22.22 %
Ipm	2.016 mA
Vpm	0.960 V
Voltage Sweep	Reverse
Sweep time	13 s
Temp	25 ℃
Irr	100 mW/cm^2
File	A20210510102050

— End of Report —

PHOTOVOLTAIC AND WIND POWER SYSTEMS QUALITY TEST CENTER, IEE, CHINESE ACADEMY OF SCIENCES

Report No: PWQC-WT-P21051021-1R

Sample code	DC2021a003
Sample S/N	FACs-1
Type	Single junction perovskite
Designated area	8.713mm^2 The designated area was offered by National Institute of Metrology, China. Test Report No. CDjc2021-10891.

Items of testing	Measurement of photovoltaic current-voltage characteristics						
Sample code	DC2021a003						
Results	Voltage Sweep	Isc (mA)	Voc (V)	Pm (mW)	E_{ff} (%)	File	
	Reverse	2.133	1.150	1.934	22.22	A20210510102050	
		Ipm (mA)	Vpm (V)	FF (%)	Area (mm^2)		
		2.016	0.960	78.89	8.713		

Supplementary Fig. 34 Certified efficiency measured by photovoltaic and wind power systems quality test center, IEE, CAS, China. No. PWQC-WT-P21051021-1R. The area of the mask was certified by National Institute of Metrology, China, No. CDjc2021-10891.

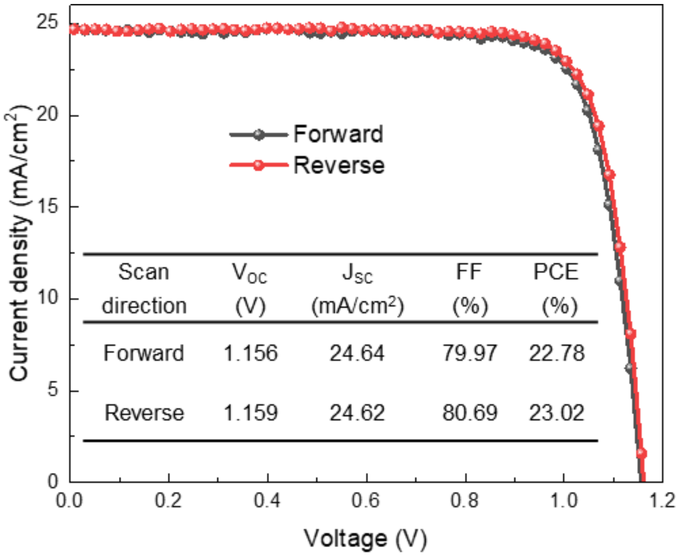

Supplementary Fig. 35 *J*-*V* curves of the NH_3-$FACsPbI_3$ PSC in both reverse and forward scan directions.

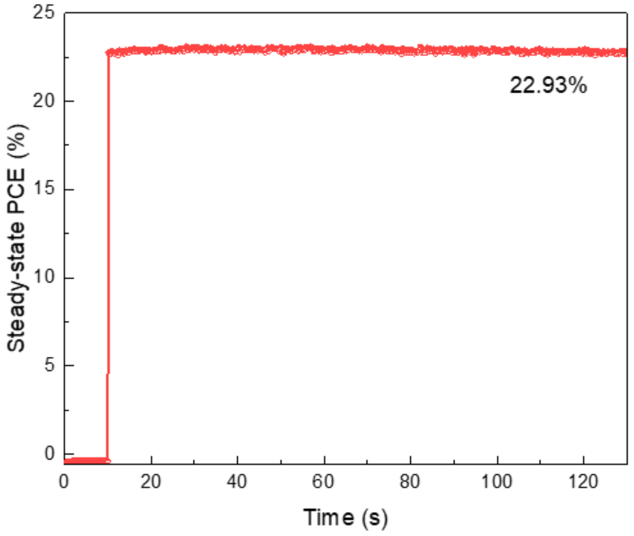

Supplementary Fig. 36 Stable power output of the NH_3-$FACsPbI_3$ PSC measured for 130 s.

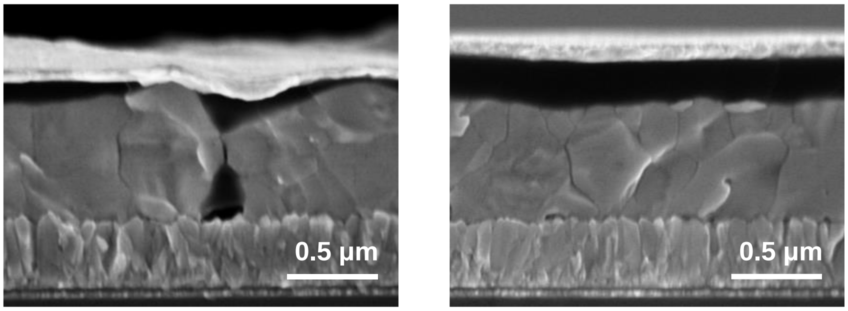

Supplementary Fig. 37 Cross-sectional SEM images of (a) Raw $FACsPbI_3$ and (b) NH_3-$FACsPbI_3$ PSCs.

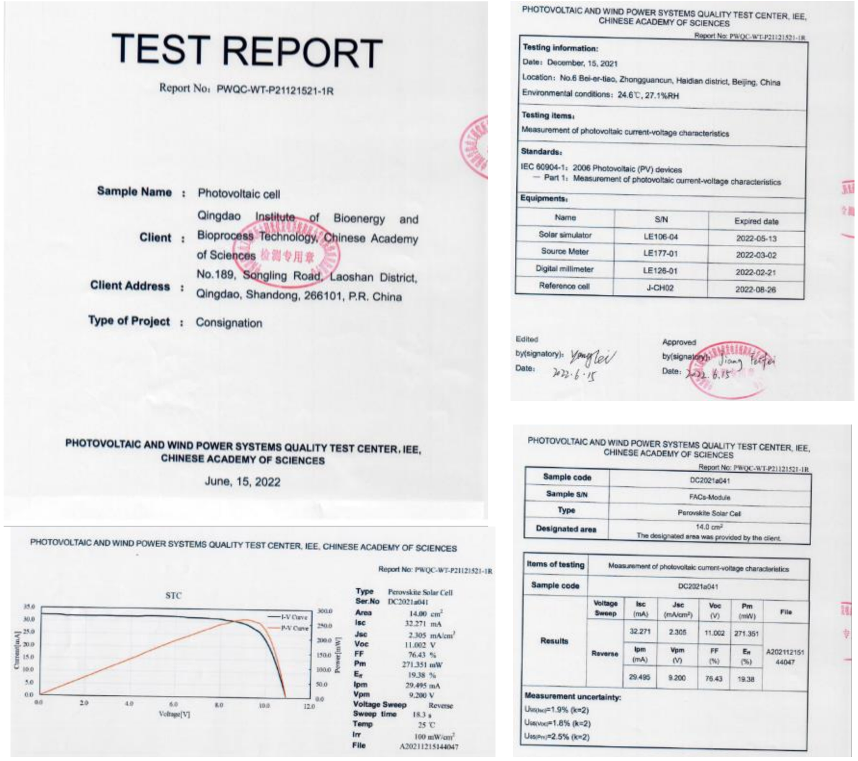

TEST REPORT

Report No: PWQC-WT-P21121521-1R

Sample Name : Photovoltaic cell

Client : Qingdao Institute of Bioenergy and Bioprocess Technology, Chinese Academy of Sciences

Client Address : No.189, Songling Road, Laoshan District, Qingdao, Shandong, 266101, P.R. China

Type of Project : Consignation

PHOTOVOLTAIC AND WIND POWER SYSTEMS QUALITY TEST CENTER, IEE, CHINESE ACADEMY OF SCIENCES

June, 15, 2022

PHOTOVOLTAIC AND WIND POWER SYSTEMS QUALITY TEST CENTER, IEE, CHINESE ACADEMY OF SCIENCES

Report No: PWQC-WT-P21121521-1R

Testing information:
Date: December, 15, 2021
Location: No.6 Bei-er-tiao, Zhongguancun, Haidian district, Beijing, China
Environmental conditions: 24.6℃, 27.1%RH

Testing items:
Measurement of photovoltaic current-voltage characteristics

Standards:
IEC 60904-1: 2006 Photovoltaic (PV) devices
— Part 1: Measurement of photovoltaic current-voltage characteristics

Equipments:

Name	S/N	Expired date
Solar simulator	LE106-04	2022-05-13
Source Meter	LE177-01	2022-03-02
Digital millimeter	LE126-01	2022-02-21
Reference cell	J-CH02	2022-08-26

Edited by(signatory): [illegible]
Date: 2022.6.15

Approved by(signatory): [illegible]
Date: 2022.6.15

PHOTOVOLTAIC AND WIND POWER SYSTEMS QUALITY TEST CENTER, IEE, CHINESE ACADEMY OF SCIENCES

Report No: PWQC-WT-P21121521-1R

STC

Current[mA]; Power[mW]; Voltage[V]; I-V Curve; P-V Curve

Type	Perovskite Solar Cell
Ser.No	DC2021a041
Area	14.00 cm^2
Isc	32.271 mA
Jsc	2.305 mA/cm^2
Voc	11.002 V
FF	76.43 %
Pm	271.351 mW
E_{ff}	19.38 %
Ipm	29.495 mA
Vpm	9.200 V
Voltage Sweep	Reverse
Sweep time	18.3 s
Temp	25 ℃
Irr	100 mW/cm^2
File	A20211215144047

PHOTOVOLTAIC AND WIND POWER SYSTEMS QUALITY TEST CENTER, IEE, CHINESE ACADEMY OF SCIENCES

Report No: PWQC-WT-P21121521-1R

Sample code	DC2021a041
Sample S/N	FACs-Module
Type	Perovskite Solar Cell
Designated area	14.0 cm^2 The designated area was provided by the client.

Items of testing	Measurement of photovoltaic current-voltage characteristics					
Sample code	DC2021a041					
Results	Voltage Sweep	Isc (mA)	Jsc (mA/cm^2)	Voc (V)	Pm (mW)	File
	Reverse	32.271	2.305	11.002	271.351	A20211215144047
		Ipm (mA)	Vpm (V)	FF (%)	E_{ff} (%)	
		29.495	9.200	76.43	19.38	

Measurement uncertainty:
U[illegible](Isc)=1.9% (k=2)
U[illegible](Voc)=1.8% (k=2)
U[illegible](Pm)=2.5% (k=2)

Supplementary Fig. 38 Certified efficiency measured by photovoltaic and wind power systems quality test center, IEE, CAS, China. No. PWQC-WT-P21121521-1R.

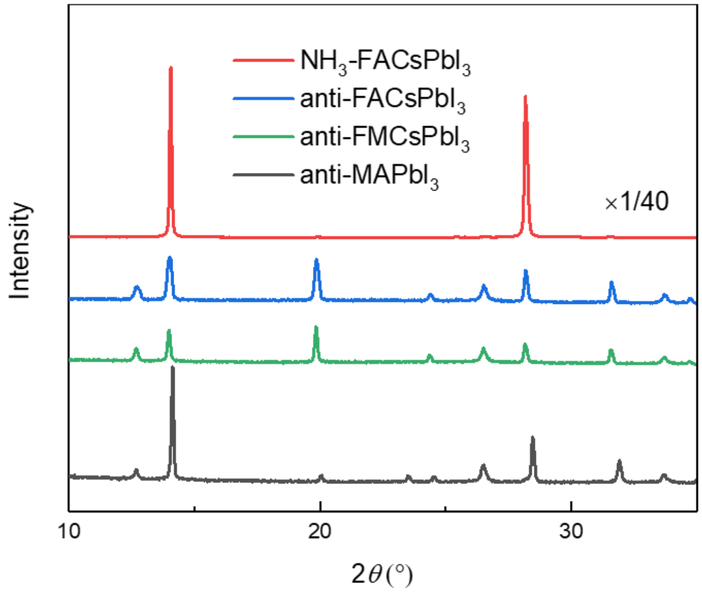

Supplementary Fig. 39 XRD patterns of the NH_3-$FACsPbI_3$, anti-$FACsPbI_3$, anti-$FMCsPbI_3$ and anti-$MAPbI_3$ devices after 320 days storage.

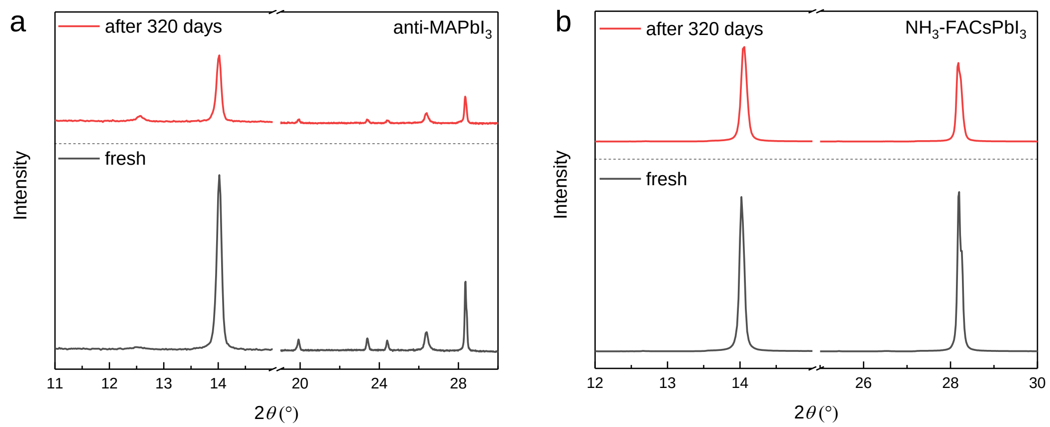

Supplementary Fig. 40 XRD patterns of (a) anti-$MAPbI_3$ and (b) NH_3-$FACsPbI_3$ devices before and after 320 days storage.

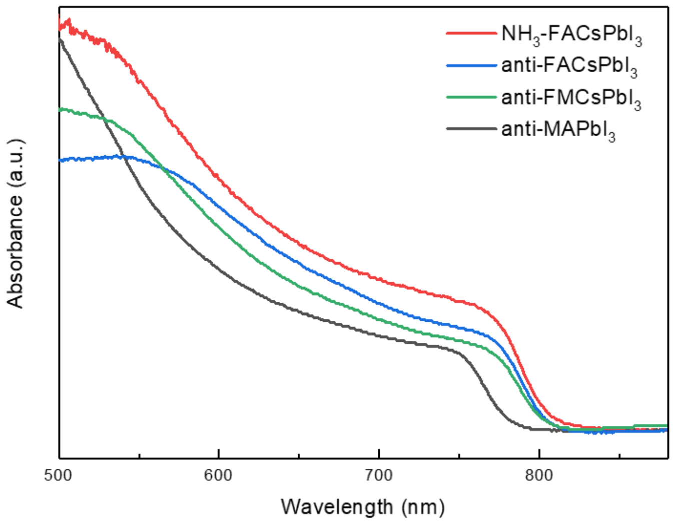

Supplementary Fig. 41 UV-vis spectra of the NH_3-$FACsPbI_3$, anti-$FACsPbI_3$, anti-$FMCsPbI_3$ and anti-$MAPbI_3$ devices after 320 days storage.

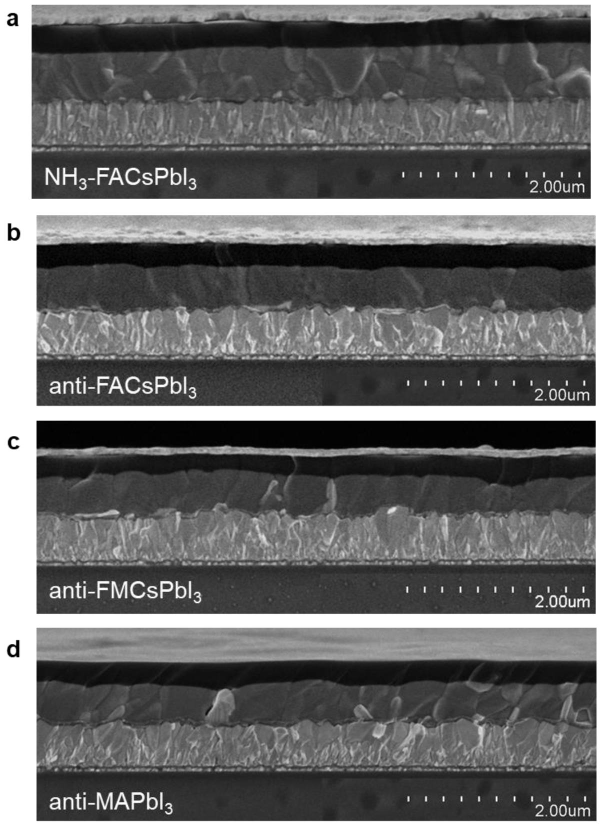

Supplementary Fig. 42 Cross-sectional SEM images of the devices after 320 days storage.

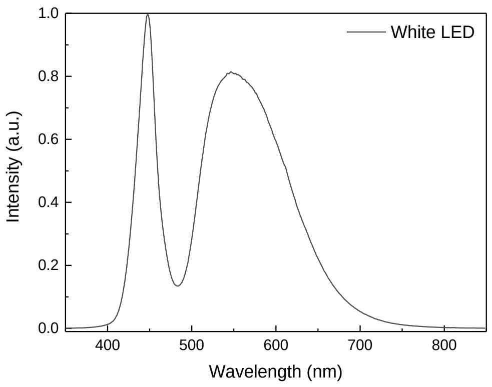

Supplementary Fig. 43 Emission spectrum of the white LEDs used in the stability test.

Supplementary Table 1 The *J*-*V* performance parameters of 50 NH_3-$FACsPbI_3$ devices at reverse scan direction.

No.	Voc (V)	Jsc (mA/cm^2)	Fill Factor (%)	PCE (%)	No.	Voc (V)	Jsc (mA/cm^2)	Fill Factor (%)	PCE (%)
1	1.14	24.31	79.06	21.96	26	1.14	24.75	76.47	21.60
2	1.17	24.48	80.39	22.94	27	1.15	24.58	76.12	21.43
3	1.15	24.41	80.10	22.57	28	1.13	24.24	76.94	21.09
4	1.16	24.63	79.98	22.92	29	1.15	24.74	77.29	21.94
5	1.16	24.09	78.81	21.93	30	1.14	23.86	77.62	21.11
6	1.16	24.53	79.87	22.77	31	1.14	24.55	77.62	21.67
7	1.16	24.10	79.05	22.08	32	1.14	24.71	80.96	22.72
8	1.16	24.54	79.93	22.70	33	1.15	24.80	80.82	22.98
9	1.14	24.76	78.44	22.20	34	1.15	24.65	81.12	23.00
10	1.15	24.61	77.55	21.95	35	1.15	24.71	80.91	22.94
11	1.15	23.75	80.16	21.83	36	1.13	24.77	80.43	22.43
12	1.14	23.94	79.90	21.84	37	1.13	24.80	80.55	22.61
13	1.16	23.36	79.42	21.54	38	1.14	24.70	80.33	22.65
14	1.16	23.90	79.92	22.20	39	1.13	24.60	80.60	22.50
15	1.16	24.52	80.94	23.10	40	1.15	24.79	77.49	22.02
16	1.16	24.30	79.17	22.33	41	1.15	24.90	78.29	22.33
17	1.12	24.21	75.91	20.67	42	1.14	24.65	79.91	22.49
18	1.13	24.42	77.47	21.47	43	1.14	24.61	78.94	22.11
19	1.13	24.14	76.59	20.86	44	1.14	24.68	78.43	22.16
20	1.14	24.27	77.48	21.51	45	1.14	24.76	79.44	22.46
21	1.14	24.23	78.04	21.65	46	1.15	24.75	77.81	22.18
22	1.13	24.96	75.15	21.18	47	1.15	24.68	80.90	23.04
23	1.13	24.74	75.67	21.15	48	1.16	24.83	80.66	23.21
24	1.15	24.64	74.33	21.02	49	1.16	24.63	80.69	23.06
25	1.14	24.65	75.05	21.05	50	1.16	24.66	81.17	23.22
