# Supplementary material for: Ammonia for post-healing of formamidinium-based Perovskite films
Source: Nat Commun. 2022 Jul 29;13:4417. doi: 10.1038/s41467-022-32047-z (PMC9338283; doi:10.1038/s41467-022-32047-z)
Supplement: Supplementary file 3 — Solar Cells Reporting Summary [file 41467_2022_32047_MOESM3_ESM.pdf]

Corresponding author(s): NCOMMS-21-47267D-Z

Solar Cells Reporting Summary

Nature Research wishes to improve the reproducibility of the work that we publish. This form is intended for publication with all accepted papers reporting the characterization of photovoltaic devices and provides structure for consistency and transparency in reporting. Some list items might not apply to an individual manuscript, but all fields must be completed for clarity.

For further information on Nature Research policies, including our data availability policy, see Authors & Referees.

Experimental design

Please check: are the following details reported in the manuscript?

1. Dimensions

Item	Response	Details
Area of the tested solar cells	☒ Yes ☐ No	Provided in the Section of Perovskite film and Device Characterization.
Method used to determine the device area	☒ Yes ☐ No	Provided in the Section of Perovskite film and Device Characterization.

2. Current-voltage characterization

Item	Response	Details
Current density-voltage (J-V) plots in both forward and backward direction	☒ Yes ☐ No	Provided in the Supplementary Information (Supplementary Figure 33).
Voltage scan conditions<br>*For instance: scan direction, speed, dwell times*	☒ Yes ☐ No	Provided in the Section of Perovskite film and Device Characterization.
Test environment<br>*For instance: characterization temperature, in air or in glove box*	☒ Yes ☐ No	Provided in the Section of Perovskite film and Device Characterization.
Protocol for preconditioning of the device before its characterization	☐ Yes ☒ No	There is no protocol for preconditioning of the device before its characterization.
Stability of the J-V characteristic<br>*Verified with time evolution of the maximum power point or with the photocurrent at maximum power point; see ref. 7 for details.*	☒ Yes ☐ No	Provided in the Section of Perovskite film and Device Characterization.

3. Hysteresis or any other unusual behaviour

Item	Response	Details
Description of the unusual behaviour observed during the characterization	☒ Yes ☐ No	Provided in the Supporting Information (Supplementary Figure 33).
Related experimental data	☒ Yes ☐ No	Provided in the Supporting Information (Supplementary Figure 33).

4. Efficiency

Item	Response	Details
External quantum efficiency (EQE) or incident photons to current efficiency (IPCE)	☒ Yes ☐ No	Provided in Figure 3b.
A comparison between the integrated response under the standard reference spectrum and the response measure under the simulator	☒ Yes ☐ No	Provided in the Section of Perovskite film and Device Characterization.
For tandem solar cells, the bias illumination and bias voltage used for each subcell	☐ Yes ☒ No	There is no tandem solar cells.

5. Calibration

Item	Response	Details
Light source and reference cell or sensor used for the characterization	☒ Yes ☐ No	Provided in the Section of Perovskite film and Device Characterization.
Confirmation that the reference cell was calibrated and certified	☒ Yes ☐ No	Provided in the Section of Perovskite film and Device Characterization.

Calculation of spectral mismatch between the reference cell and the devices under test

- [x] Yes
- [ ] No

Provided in the Section of Perovskite film and Device Characterization.

6. Mask/aperture

Size of the mask/aperture used during testing

- [x] Yes
- [ ] No

Provided in the Section of Perovskite film and Device Characterization.

Variation of the measured short-circuit current density with the mask/aperture area

- [ ] Yes
- [x] No

We have not measured short-circuit current density with aperture area.

7. Performance certification

Identity of the independent certification laboratory that confirmed the photovoltaic performance

- [x] Yes
- [ ] No

Provided in Supplementary Information (Supplementary Figure 32, 38)

A copy of any certificate(s)
Provide in Supplementary Information

- [x] Yes
- [ ] No

Provided in Supplementary Information (Supplementary Figure 32, 38)

8. Statistics

Number of solar cells tested

- [x] Yes
- [ ] No

Provided in Supplementary Information (Supplementary Table 1).

Statistical analysis of the device performance

- [x] Yes
- [ ] No

Provided in Supplementary Information (Supplementary Table 1).

9. Long-term stability analysis

Type of analysis, bias conditions and environmental conditions
For instance: illumination type, temperature, atmosphere humidity, encapsulation method, preconditioning temperature

- [x] Yes
- [ ] No

Provided in Figure 3e, f.
